# Digital Mindfulness Training for Burnout Reduction in Physicians: Clinician-Driven Approach

**DOI:** 10.2196/63197

**Published:** 2025-01-24

**Authors:** Lia Antico, Judson Brewer

**Affiliations:** 1 Brown University Department of Behavioral and Social Sciences Providence, RI United States

**Keywords:** burnout, anxiety, empathy fatigue, physician, mindfulness, digital therapeutics, app, smartphone, podcast, compassion, health care provider, training, physician burnout, cynicism, efficacy, treatment, meditation, chronic, workplace stress, digital health, mHealth, mobile phone

## Abstract

**Background:**

Physician burnout is widespread in health care systems, with harmful consequences on physicians, patients, and health care organizations. Mindfulness training (MT) has proven effective in reducing burnout; however, its time-consuming requirements often pose challenges for physicians who are already struggling with their busy schedules.

**Objective:**

This study aimed to design a short and pragmatic digital MT program with input from clinicians specifically to address burnout and to test its efficacy in physicians.

**Methods:**

Two separate nonrandomized pilot studies were conducted. In the first study, 27 physicians received the digital MT in a podcast format, while in the second study, 29 physicians and nurse practitioners accessed the same training through a free app-based platform. The main outcome measure was cynicism, one dimension of burnout. The secondary outcome measures were emotional exhaustion (the second dimension of burnout), anxiety, depression, intolerance of uncertainty, empathy (personal distress, perspective taking, and empathic concern subscales), self-compassion, and mindfulness (nonreactivity and nonjudgment subscales). In the second study, worry, sleep disturbances, and difficulties in emotion regulation were also measured. Changes in outcomes were assessed using self-report questionnaires administered before and after the treatment and 1 month later as follow-up.

**Results:**

Both studies showed that MT decreased cynicism (posttreatment: 33% reduction; *P*≤.04; *r*≥0.41 and follow-up: 33% reduction; *P*≤.04; *r*≥0.45), while improvements in emotional exhaustion were observed solely in the first study (25% reduction, *P*=.02, *r*=.50 at posttreatment; 25% reduction, *P*=.008, *r*=.62 at follow-up). There were also significant reductions in anxiety (*P*≤.01, *r*≥0.49 at posttreatment; *P*≤.01, *r*≥0.54 at follow-up), intolerance of uncertainty (*P*≤.03, *r*≥.57 at posttreatment; *P*<.001, *r*≥0.66 at follow-up), and personal distress (*P*=.03, *r*=0.43 at posttreatment; *P*=.03, *r*=0.46 at follow-up), while increases in self-compassion (*P*≤.02, *r*≥0.50 at posttreatment; *P*≤.006, *r*≥0.59 at follow-up) and mindfulness (nonreactivity: *P*≤.001, *r*≥0.69 at posttreatment; *P*≤.004, *r*≥0.58 at follow-up; nonjudgment: *P*≤.009, *r*≥0.50 at posttreatment; *P*≤.03, *r*≥0.60 at follow-up). In addition, the second study reported significant decreases in worry (*P*=.04, *r*=0.40 at posttreatment; *P*=.006, *r*=0.58 at follow-up), sleep disturbances (*P*=.04, *r*=0.42 at posttreatment; *P*=.01, *r*=0.53 at follow-up), and difficulties in emotion regulation (*P*=.005, *r*=0.54 at posttreatment; *P*<.001, *r*=0.70 at follow-up). However, no changes were observed over time for depression or perspective taking and empathic concern. Finally, both studies revealed significant positive correlations between burnout and anxiety (cynicism: *r*≥0.38; *P*≤.04; emotional exhaustion: *r*≥0.58; *P*≤.001).

**Conclusions:**

To our knowledge, this research is the first where clinicians were involved in designing an intervention targeting burnout. These findings suggest that this digital MT serves as a viable and effective tool for alleviating burnout and anxiety among physicians.

**Trial Registration:**

ClinicalTrials.gov NCT06145425; https://clinicaltrials.gov/study/NCT06145425

## Introduction

### Background

Burnout is an occupational phenomenon that is highly prevalent among physicians globally. While the rates have increased because of the COVID-19 pandemic, this was only the acceleration of an existing trend over the last decade [1-4]. In a 2021 survey of 2500 US physicians, 62.8% reported at least 1 symptom of burnout, compared with 38.2% in 2020, 43.9% in 2017, 54.4% in 2014, and 45.5% in 2011 [4].

Defined by the World Health Organization as “a syndrome resulting from chronic workplace stress that has not been managed successfully,” burnout is characterized by 3 dimensions: emotional exhaustion, cynicism, and reduced personal accomplishment [5]. *Emotional exhaustion* includes feeling overwhelmed by work demands and experiencing depletion of emotional and physical resources. *Cynicism* represents the interpersonal aspect of burnout and refers to callous and detached attitudes toward one’s job and people. *Reduced personal accomplishment* refers to poor professional self-esteem and efficacy [6].

The consequences of burnout on physicians, patients, and health care organizations are significant and far reaching. A large number of studies showed that physicians reporting high rates of burnout are also those who report high levels of anxiety [7], sleep disturbances [8-10], depression, and substance abuse [11,12]. Suicide rates among physicians are twice as high as those in the general public [13]. The emotional toll is so high that in a 2020 survey, 31% of physicians planned to reduce their work hours, and 24% of physicians intended to leave their job within 2 years [14]. An estimation of the direct costs of physician turnover and reduced clinical work is approximately US $4.6 billion each year in the United States [15]. Meta-analyses found that physician burnout is also associated with increased risk of patient safety incidents and malpractice claims, poorer quality due to low professionalism, and diminished and ineffective communication between physicians and patients [16,17]. Indirect costs from medical errors, reduced patient satisfaction, damage to the organization’s reputation, and decreased patient loyalty raise the burnout bill to approximately US $7.6 million annually per employed physician [15].

### Associated Factors of Physician Burnout

Physician burnout is complex and multifactorial. Burnout among physicians arises from both institutional and individual factors that create an imbalance between job demands and available resources. For instance, high workload and increased administrative tasks (eg, the electronic medical record systems) lead to overworking and deprioritizing self-care. A study involving 7288 US physicians found that for every additional hour worked per week beyond 51.8 hours, burnout symptoms rose by approximately 2% [18]. Similarly, research on 1490 US oncologists revealed that each additional hour spent working at home led to a 2% rise in burnout [2]. Another major contributor to burnout is the loss of flexibility, autonomy, and control that occurs when health care organizations and leadership fail to support individual work goals. This leaves physicians excluded from decisions about patient visit durations and treatment approaches [19].

It may not be a coincidence that the term “burnout” was first used in 1974 to describe the exhaustion observed in people in “healing professions” [20]. Indeed, physicians are constantly exposed to highly stressful and demanding situations that are intrinsic to the clinical practice. Many aspects of medicine are uncertain and ambiguous. For example, diagnoses are often unclear, the natural course of illness is unpredictable, responses of patients to treatments vary, and the clinical information is complex. A growing body of literature indicates that physicians’ reactions to uncertainty and their ability to tolerate it can predict stress [21]. Cross-sectional studies reported negative correlations between intolerance of uncertainty and well-being of physicians [22,23]. In particular, physicians with a high intolerance for uncertainty are more likely to experience higher rates of burnout and anxiety, make more referrals, and report lower job satisfaction [24]. However, to date, no studies have directly tested the effects of interventions aimed at reducing physician burnout on intolerance of uncertainty.

While alleviating patients’ distress is among the main reasons why many physicians enter the healing professions, caring for the sick is not without consequences. Repeated exposure to illness, death, or social inequities may cause empathic distress that leads to empathy fatigue (sometimes called inappropriately compassion fatigue [25]) and burnout.

Empathy is defined as the capacity to understand and share others’ emotions, feelings, and mental states by imagining or taking another person’s perspective [25]. Clinical empathy is an essential element of quality care, associated with improved patient satisfaction; increased adherence to treatment; better surgical recovery; shorter hospital stays [26-30]; and increased physician health, well-being, and professional satisfaction [31,32]. However, overempathizing can lead to being overwhelmed by the patient’s distress, which might result in withdrawal and contribute to empathy fatigue. Conversely, maintaining a professional perspective with compassionate concern allows physicians to engage with the patient’s distress without taking it personally, fostering stronger connections while reducing the risk of burnout.

### Interventions for Physician Burnout

Combating burnout is a shared responsibility between the institution and the individual. Effective initiatives have been developed and implemented at both levels [33]. Organization-directed interventions target workload, foster communication between members of the health care system, and cultivate a sense of team cohesion and job control [33,34]. Individual-focused approaches aim to decrease perceived stress, increase resilience to stressful work environments, and enhance work engagement [33-35]. In particular, this paper focuses on the tangible support that could be provided to physicians at the individual level.

### Mindfulness Training

Several studies have reported that mindfulness training (MT) is one of the most studied individual-focused interventions for reducing stress and enhancing well-being among physicians [33,36-40]. Mindfulness refers to being fully aware and present in the current moment, on purpose and nonjudgmentally, of thoughts, feelings, and sensations [41]. By fostering self-awareness and attitudes of openness and “nonjudgment,” MT facilitates acceptance of the challenging components of experiences and reduces emotional reactivity [42], both of which are crucial in health care. Most studies have observed that MT reduces rumination, worry, and emotional reactivity among health care providers [40,43]. For instance, nonreactivity to inner experiences has been inversely associated with perceived stress in health care providers [44].

In addition, a meta-analysis found that MT also increases self-compassion in health care providers, with moderate to high effect sizes [45]. Self-compassion involves being touched by one’s own distress and being motivated to alleviate it with kindness and understanding [46,47]. It has been theorized that self-compassion is pertinent to physicians’ well-being at work and care for patients [48], as it was inversely associated with burnout and empathy fatigue among health care professionals [49-52]. For example, it has been found that physicians who were more self-compassionate felt less exhausted due to work demands and experienced greater work engagement and satisfaction with their professional life [53].

These findings suggest that MT may be promising as an intervention for physician burnout. Different formats have been used in clinical settings [54]; some of them are intensive, including weekly groups sessions of 2.5 hours, home practices of 45 minutes for 8 weeks, and a 7-hour retreat [39]. Therefore, shorter and more practical formats are needed to avoid inadvertently contributing to overload. For example, in a study, 44% of health care professionals randomized to an MT dropped the program because of “lack of time” [55]. Even when health care providers are willing to attend MT, work conflicts can often arise and affect attendance and home practice adherence [35]. In a qualitative study, physicians confirmed the role of mindfulness interventions in the development of self-awareness and highlighted the importance of adapting the course timing and materials to meet their schedule, thereby improving participation and adherence [56].

Furthermore, engaging physicians in the design and implementation of interventions might increase their sense of control and engagement (“user-centered design”) [57] and might make the interventions more feasible and tailored to their specific needs. However, to our knowledge, no studies have yet addressed this issue. This study is one of the few that involve clinicians in designing an intervention to reduce physician burnout. Successively, we conducted 2 independent single-arm studies and assessed the impact of digital MT training on physician burnout. In study 1, the intervention was delivered in a podcast format, while in study 2, it was delivered via a free app-based platform.

## Study 1

### Overview

To develop a short and tailored intervention, we used a user-centered design approach by involving clinicians and focusing on their needs in each phase of the development process. After iterative content development, we delivered the digital intervention in the format of an audio podcast, with the reasoning that content could be consumed during one’s commute to and from work and, thus, not add more to the physician’s workload. The primary goal of this single-arm study was to examine whether the intervention reduces cynicism (ie, one dimension of burnout) in physicians. The secondary goal was to assess its effects on other variables, such as emotional exhaustion (ie, another dimension of burnout), anxiety, depression, mindfulness (ie, the aspects of nonreactivity and nonjudging of inner experiences), intolerance of uncertainty, empathy (ie, the dimensions of empathic concern, perspective taking, and personal distress), and self-compassion. Finally, we explored the relationship between the 2 dimensions of burnout (ie, cynicism and emotional exhaustion) and the other secondary outcomes.

We evaluated the following hypotheses:

- Hypothesis 1: there will be a main effect of time on cynicism. Physicians will report lower scores for cynicism at postintervention assessments compared to baseline.
- Hypothesis 2: there will be a main effect of time on the other variables. Physicians will report lower scores for emotional exhaustion, anxiety, depression, intolerance of uncertainty, and personal distress and higher scores for nonreactivity, nonjudging of inner experiences, empathic concern, perspective taking, and self-compassion at postintervention assessments compared to baseline.
- Hypothesis 3: cynicism and emotional exhaustion will positively correlate with anxiety.

### Methods

#### Ethical Considerations

The study was approved by the Brown University Institutional Review Board (protocol 2022003296) and was conducted in accordance with the Declaration of Helsinki for experiments involving human subjects. Eligible individuals were directed to the informed consent form on Qualtrics (Qualtrics International, Inc). After enrolling by selecting “yes” (a waiver of documentation of informed consent was obtained), they were redirected to complete the web-based survey. Participants were compensated with a US $25 Amazon gift card for the completion of each survey (US $75 for 3 surveys). All collected data were anonymized, with each participant being assigned a unique alphanumeric code. For statistical analyses, the final database contained only these alphanumeric codes to ensure privacy.

#### Participants

Physicians were recruited using university hospital mailing lists and flyer advertisements targeting physicians who were interested in testing whether a short training program was effective at reducing burnout. We used the following text in the recruitment message: “Would you like to help us test a short mindfulness-based training to reduce burnout? If you are a physician and are interested in helping us learn about burnout, please contact us.”

Inclusion criteria were as follows: direct patient interaction; currently employed as a physician; fluency in English; endorsed willingness to listen to a mindfulness audio course for 15 minutes per day for 7 days; and endorsed willingness to complete 3 web-based surveys, before and after the completion of the training and 1 month later as follow-up. The only exclusion criteria were changing the dose of psychotropic medication in the past 6 weeks and prior exposure to this specific podcast or its content.

#### Intervention

The mindfulness-based training teaches physicians how to acknowledge and support patients’ suffering without becoming overwhelmed by it. It also teaches them to identify and work with empathy fatigue habits loops as well as anxious and worry thoughts that contribute to burnout (eg, not being able to stop thinking about a problem, worrying about patients, and taking work home). Specifically, this program trains awareness and curiosity by targeting fundamental learning processes, such as reinforcement learning, to alter entrenched habits [58]. Previous studies have demonstrated that these changes can lead to significant, clinically relevant effects [58-61].

This experiential education is delivered via an audio podcast, which consists of 7 modules of brief didactic and experience-based MT, each approximately 15 minutes long, and a summary module at the end. Each module includes real-world vignettes and practical mindfulness exercises designed to help identify habit loops in clinical practice and work-related tasks and to provide strategies for breaking these unhelpful loops (Textbox 1). We also provided links to guided meditations, including noting, breathing, grounding practices, as well as curiosity and loving-kindness meditations. Although participants were encouraged to practice these guided meditations, they were optional and not mandatory for participation in this study.

**Overview of the audio course: burnout to resilience themes and content.**

**Module 1: goals, empathy fatigue, and unhelpful habits**

- Set goals and introduce how important it is to be empathetic with patients in their recovery and its limits. Help physicians reflect on the different coping strategies used to protect themselves from patients’ distress that become habits and contribute to exhaustion. It discusses specific strategies for stepping out of unhelpful habit loops.

**Module 2: empathy versus compassion**

- Addresses specifically the difference between empathy and compassion in patterns of activations in the body and mind, and it introduces compassion as a new way to feel patients’ distress without being emotionally exhausted by it.

**Module 3: intrusive thoughts**

- Explains that intrusive thoughts are distracting and can cause great distress. This module also provides strategies for handling them and coming back to the present moment.

**Module 4: self-judgment and kindness**

- Describes how the mind can be caught up in the loop of self-judgments that contribute to exhaustion by getting in the way of doing one’s own tasks and interacting with patients. This module also focuses on how kindness can be a detox of self-judgment.

**Module 5: stress and anxiety**

- Discusses why the brain does not like uncertainty and how this causes stress and anxiety. This module also provides strategies to change habit patterns and reduce stress and anxiety.

**Module 6: emotional contagion and curiosity**

- Introduces emotional contagion and its impact on the interactions with patients and colleagues. This module also explains how curiosity can be used as a vaccine against emotional contagion.

**Module 7: bias in the diagnosis and treatment of pain**

- Describes the complexity of pain, including factors and biases that influence the diagnosis and treatment of pain in patients, and provides strategies for recognizing and dealing with those biases.

The content of this intervention was developed using a user-centered design approach, an iterative design process in which designers focus on the users and their needs in each phase of the design process. On the basis of the extant literature and interviews of the physicians, we identified common individual empathy fatigue “habits” that contribute to burnout [62-64]. Scripts were written by a physician with >20 years of clinical and mindfulness practice and >10 years of experience in developing MT programs [59-61]. We developed a “minimum viable product” consisting of script-based content that users read, tried out, and commented. We refined the audio course and tested its suitability for a wide range of physicians and other health care providers by collecting feedback from 40 clinicians in 2 rounds. In round 1, a total of 10 physicians from different subspecialties were emailed a script, instructions, and a link to a feedback form. The feedback form included open-ended questions about what participants learned, what was helpful in their clinical practice and personal life, and any confusion or difficulties with understanding and completing home practices and slider questions (ranging from 0 to 100) about how well the module met their needs and those of their colleagues, the usefulness of the real-life stories shared, and the clarity of the home practice instructions. Once each module was completed (as indicated by a completed feedback form), the next module was emailed to the pilot tester. Feedback was collated, and common themes were extracted for training refinement. Vignettes from this round of pilot testers were also incorporated for story-based learning [65]. In round 2, a total of 30 physicians and other clinicians were recruited to repeat this process to confirm content fit and gather additional real-world vignettes for potential incorporation.

#### Intervention Orientation and Engagement

Participants were instructed to complete the modules at their own pace, with a maximum of 1 module per day. The project director reached out with check-in messages every 15 days from the start of the training to address technical difficulties and encourage continued engagement. During these check-ins, participants were asked about their experience with the podcast since the last contact. If participants expressed difficulties, efforts were made to resolve the problem.

#### Measures

Primary and secondary outcomes were assessed at 3 time points: baseline (before training), postintervention (after training completion), and follow-up (1 month after training completion).

#### Primary Outcome

Cynicism, a key dimension of burnout, was measured using a single item from the Maslach Burnout Inventory. This approach is supported by research from West et al [66,67], which demonstrated the validity of using a single item in relation to the full 22-item questionnaire. Participants rated how often they felt “more callous toward people since starting this job” on a 7-point scale (0=“never” and 6=“every day”). Higher scores indicate greater cynicism [68].

#### Secondary Outcomes

*Emotional exhaustion* was measured using a single Maslach Burnout Inventory item. Participants rated how often they felt “burned out from their work” on a 7-point scale, with higher scores indicating greater exhaustion [68].

Anxiety was measured using the *Generalized Anxiety Disorder-7* on a 4-point scale (0=“not at all” and 3=“nearly every day”). Scores range from 0 to 21, with higher scores indicating more severe anxiety. Total scores of 5 to 9, 10 to 14, and ≥15 correspond to cutoff points of mild, moderate, and severe anxiety, respectively [69].

Depression was measured using the *Patient Health Questionnaire-2* assessing depressive symptoms over the past 2 weeks. Participants rated each item on a 4-point scale (0=“not at all” and 3=“nearly every day”), with higher scores indicating more severe symptoms [70].

Intolerance *of uncertainty* was assessed by the *Intolerance of Uncertainty scale-12* measuring intolerance of uncertainty with 12 items rated on a 5-point scale (1=“not at all” to 5=“entirely”). Total scores range from 12 to 60, with higher scores indicating greater intolerance [71].

Self-compassion was measured by the *Self-Compassion Scale-Short Form* assessing self-compassion using 12 items rated on a 5-point scale (1=“almost never” to 5=“almost always”). Total scores range from 12 to 60, with higher scores indicating greater self-compassion [72].

Mindfulness was assessed using the *Five Facet Mindfulness Questionnaire* measuring nonreactivity and nonjudgment, with subscales rated on a 5-point scale (1=“never true” to 5=“always true”). Nonreactivity total scores range from 7 to 35, with higher scores indicating a greater ability to not react to or get caught up in thoughts or emotions. Nonjudgment total scores range from 8 to 40, with higher scores indicating a greater ability to not judge or criticize thoughts or emotions [73].

Empathy was assessed using the *Interpersonal Reactivity Index* that measured empathy using 4 subscales (empathic concern, perspective taking, personal distress, and fantasy). Items were rated on a 5-point scale (0=“does not describe me” to 4=“describes me well”). Each subscale consists of 7 items, and total scores range from 0 to 28, with higher scores indicating greater empathy across subscales. The fantasy subscale was excluded as not relevant [74].

#### Statistical Analysis

Statistical analyses were conducted in R (version 4.2.2; R Foundation for Statistical Computing). Friedman ANOVA, a nonparametric test, was used to analyze the overall change in cynicism scores (primary outcome) and the other scores (secondary outcomes) at the 3 time points due to the data having a nonnormal distribution. Post hoc analyses between the individual time points were analyzed using Wilcoxon signed rank tests and were corrected for multiple comparisons using the Benjamini-Hochberg procedure. The relationship between the dimensions of burnout and the other variables was evaluated using the Spearman rank correlation coefficient. Effect sizes were reported as Kendall W= χ^2^/N(K−1) for Friedman tests and as Pearson *r*=z/sqrt (N) for pairwise Wilcoxon signed rank tests. Kendall W and Pearson *r* use Cohen criteria where 0.1 to <0.3 is a small effect, 0.3 to <0.5 is a medium effect, and ≥0.5 is a large effect [75].

### Results

#### Participants

**Figure 1:**
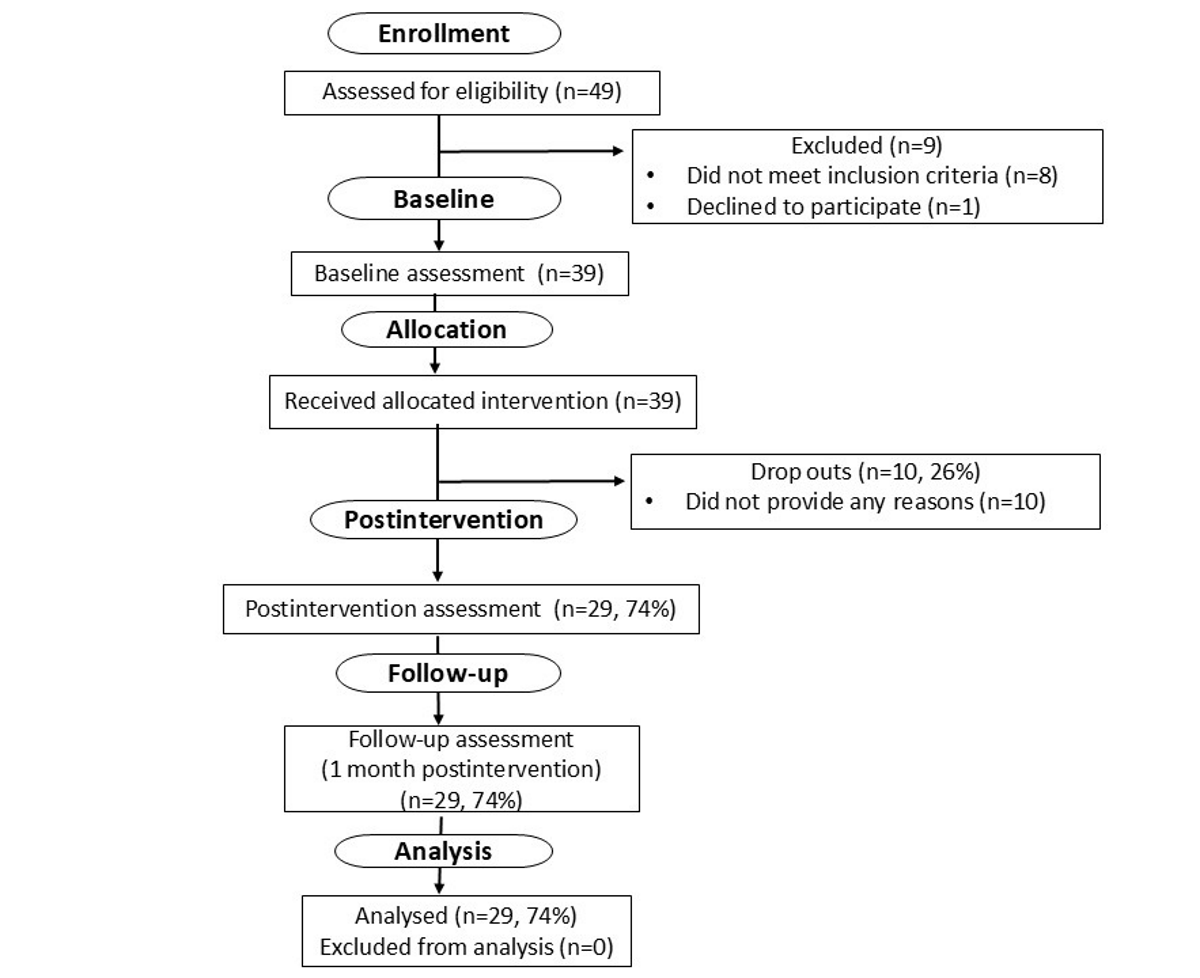
CONSORT (Consolidated Standards of Reporting Trials) flow diagram of study procedures of study 1.

A total of 40 physicians replied to our recruitment message, and 32 (80%) participants met eligibility criteria and consented to participate between August 2022 and February 2023. Out of this group who received the allocated intervention, of the 32 physicians, 27 (84%) completed assessments at all 3 time points and were included in the final analysis (Figure 1). The participant population comprised 20 women and 7 men who worked in health care for an average of 15 (SD 9.76) years. The average age was 50.6 (SD 9.74) years (Table 1).

**Table 1:** Demographic characteristics of study 1.

			Participants (N=27)
Age (y), mean (SD)			50.6 (9.74)
**Gender, n (%)**			
	Man	7 (26)	
	Woman	20 (74)	
**Race and ethnicity, n (%)**			
	Asian	5 (19)	
	Black or African American	1 (4)	
	Hispanic, Latinx, or Spanish	0 (0)	
	White	21 (77)	
**Work status, n (%)**			
	Employed full time	22 (82)	
	Employed part time	5 (8)	
Years working in health care, mean (SD)			15 (9.76)
**Medical specialty** **, n (%)**			
	Emergency medicine	2 (7)	
	Pediatric emergency medicine	3 (12)	
	Pediatric hematology oncology	2 (7)	
	Pulmonary and critical care medicine	2 (7)	
	Family medicine	6 (22)	
	Internal medicine	5 (19)	
	Neurology	1 (4)	
	Neurocritical care	1 (4)	
	Psychiatry	2 (7)	
	Infectious disease	1 (4)	
	Obstetrics-gynecology	2 (7)	

#### Primary and Secondary Outcomes

The average time taken by participants to complete the program was 30 (SD 16) days.

Table 2 shows the outcome scores at each assessment point compared with baseline.

Friedman ANOVA tests indicated significant changes over time for cynicism (*χ²_2_*=6.96; *P*=.03; W=0.13), emotional exhaustion (*χ²*_2_=12.6; *P*=.002; W=0.23), anxiety (*χ²*_2_=10.7; *P*=.005; W=0.20), intolerance of uncertainty (*χ²*_2_=12.1; *P*=.002; W=0.22), self-compassion (*χ²*_2_=9.88; *P*=.007; W=0.18), nonreactivity (*χ²*_2_=16.7; *P*<.001; W=0.31), and nonjudgment of inner experiences (*χ²*_2_=19.5; *P*<.001; W=0.36) and personal distress (a subdimension of empathy; *χ^2^*_2_=6.74; *P*=.03; W=0.13). No significant change was observed across the 3 time points for the other subdimensions of empathy, such as perspective taking (*χ^2^*_2_=5.75; *P*=.06; W=0.106) and empathic concern (*χ^2^*_2_=90.3; *P*=.86; W=0.006), and for depression (*χ^2^*_2_=1.72; *P*=.42; W=0.032).

**Figure 2:**
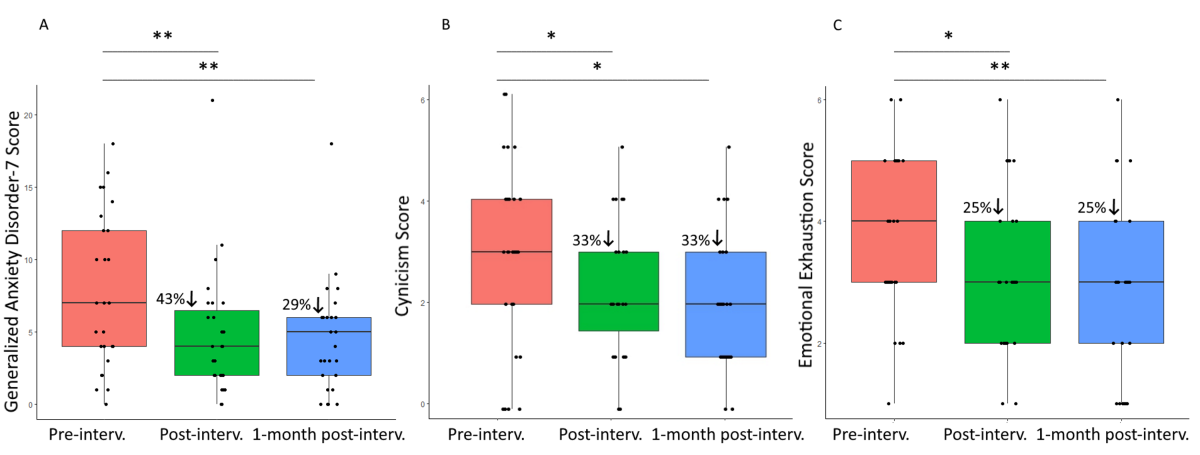
Box and whisker plots at baseline, at postintervention, and 1 month after intervention completion for (A) Generalized Anxiety Disorder-7 scores, (B) cynicism scores, and (C) emotional exhaustion scores from the Maslach Burnout Inventory. *P=.05, **P=.01.

Post hoc Wilcoxon signed rank tests revealed 33% reduction of cynicism from baseline to postintervention (W_26_=134; *P*=.04; *r*=0.41) and 1 month later (W_26_=140; *P*=.04; *r*=0.47), 25% reduction of emotional exhaustion at both points (postintervention: W_26_=116; *P*=.02; *r*=0.50 and follow-up: W_26_=154; *P*=.008; *r*=0.62), and 43% reduction of anxiety at postintervention (W_26_=244; *P*=.004; *r*=0.62) and 29% one month later (W_26_=228; *P*=.01; *r*=0.54; Figure 2).

**Figure 3:**
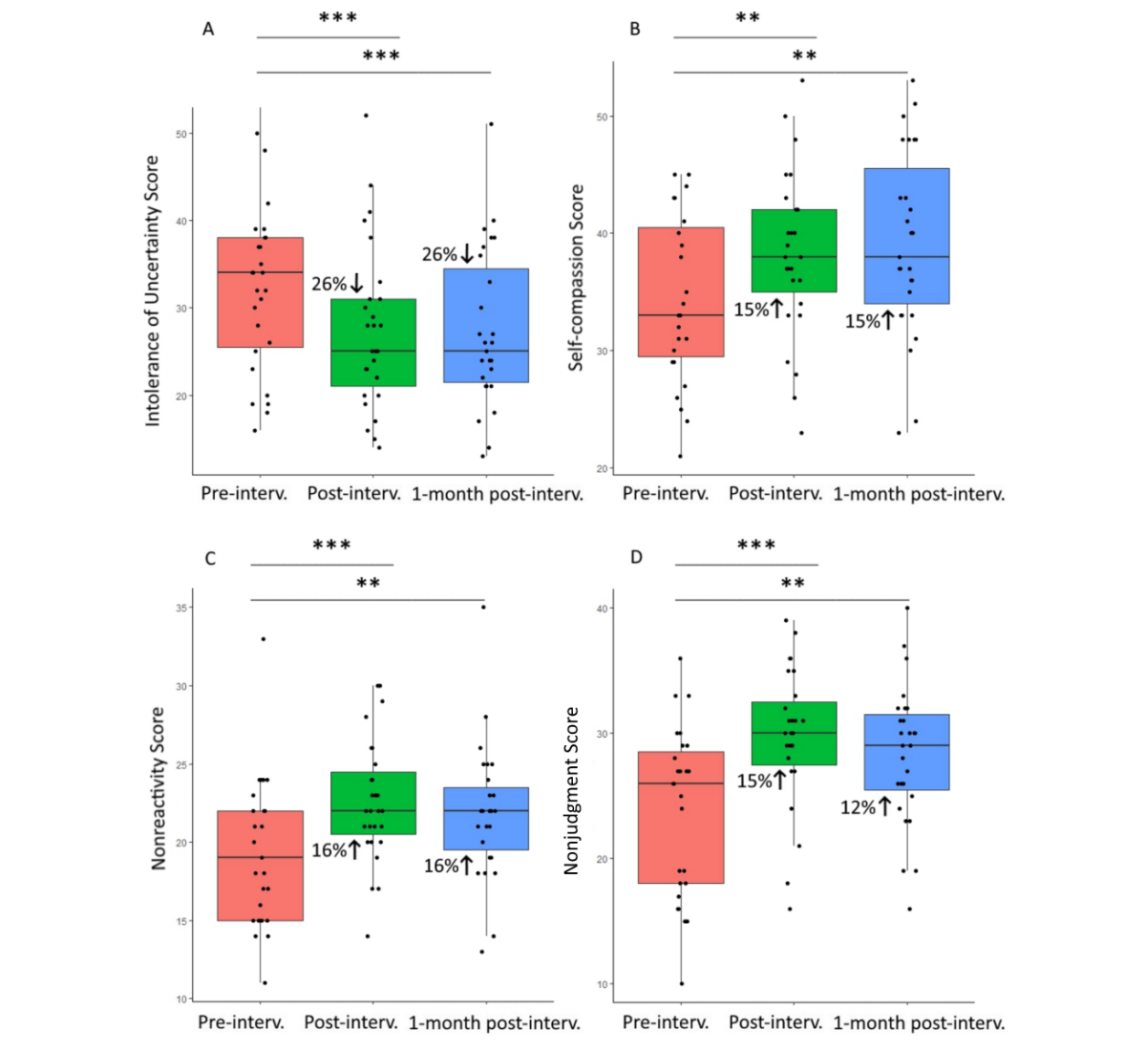
Box and whisker plots at baseline, at postintervention, and 1 month after intervention completion for (A) intolerance of uncertainty scores, (B) self-compassion scores, (C) nonreactivity, and (D) nonjudgment of inner experiences scores. *P=.05, **P=.01, ***P=.001.

Wilcoxon signed rank tests revealed 26% reduction of intolerance of uncertainty at postintervention (W_26_=314; *P*<.001; *r*=0.67) and 1 month after the end of the intervention (W_26_=242; *P*<.001; *r*=0.70), 15% increase of self-compassion at both points (postintervention: W_26_=73.5; *P*=.02; *r*=0.50 and follow-up: W_26_=36; *P*=.006; *r*=0.59), 16% increase of nonreactivity (postintervention: W_26_=22.5; *P*=.001; *r*=0.69 and follow-up: W_26_=50; *P*=.004; *r*=0.58), and 15% increase of nonjudgment of inner experiences at postintervention (W_26_=5; *P*<.001; *r*=0.81) and 12% increase 1 month later (W_26_=47; *P*=.003; *r*=0.60; Figure 3).

**Table 2:** Descriptive statistics for primary and secondary outcomes (N=27 physicians).

		Values, mean (SD)	Values, median (IQR)	*P* value	Effect sizes (*r*)
**Cynicism**					
	Baseline	2.93 (1.77)	3 (2)	—^a^	—
	Postintervention	2.26 (1.26)	2 (1.5)	—	—
	1-mo postintervention	2.07 (1.38)	2 (2)	—	—
	Δ^b^ at postintervention (%)	−0.67 (−23)	−1 (−33)	.04	0.41
	Δ at 1-mo postintervention (%)	−0.86 (−29)	−1 (−33)	.04	0.47
**Emotional exhaustion**					
	Baseline	3.89 (1.31)	4 (2)	—	—
	Postintervention	3.22 (1.31)	3 (2)	—	—
	1-mo postintervention	2.93 (1.41)	3 (2)	—	—
	Δ at postintervention (%)	−0.67 (−17)	−1 (−25)	.02	0.50
	Δ at 1-mo postintervention (%)	–0.96 (−25)	−1 (−25)	.08	0.62
**GAD-7^c^**					
	Baseline	7.63 (5.25)	7 (8)	—	—
	Postintervention	4.59 (4.45)	4 (4.5)	—	—
	1-mo postintervention	4.67 (3.85)	5 (4)	—	—
	Δ at postintervention (%)	−3.04 (−40)	−3 (−43)	.004	0.62
	Δ at 1-mo postintervention (%)	−2.96 (−39)	−2 (−29)	.01	0.54
**PHQ-2** ^d^					
	Baseline	0.56 (0.70)	0 (1)	—	—
	Postintervention	0.63 (1.12)	0 (1)	—	—
	1-mo postintervention	0.52 (0.80)	0 (1)	—	—
	Δ at postintervention (%)	−0.07 (−12)	0 (0)	.99	0.03
	Δ at 1-mo postintervention (%)	−0.04 (−7)	0 (0)	.97	0.11
**Intolerance of uncertainty**					
	Baseline	32.6 (10)	34 (12.5)	—	—
	Postintervention	27.4 (9.31)	25 (10)	—	—
	1-mo postintervention	27.4 (8.97)	25 (13)	—	—
	Δ at postintervention (%)	−5.2 (16)	−9 (−26)	<.001	0.67
	Δ at 1-mo postintervention (%)	−5.2 (16)	−9 (−26)	<.001	0.70
**Self-compassion**					
	Baseline	34.3 (7)	33 (11)	—	—
	Postintervention	38.1 (6.97)	38 (7)	—	—
	1-mo postintervention	39.2 (7.87)	38 (11.5)	—	—
	Δ at postintervention (%)	3.8 (11)	5 (15)	.02	0.50
	Δ at 1-mo postintervention (%)	4.9 (14)	5 (15)	.006	0.59
**Nonreactivity**					
	Baseline	19.3 (4.65)	19 (7)	—	—
	Postintervention	22.6 (3.90)	22 (4)	—	—
	1-mo postintervention	21.9 (4.26)	22 (4)	—	—
	Δ at postintervention (%)	3.3 (17)	3 (16)	.001	0.69
	Δ at 1-mo postintervention (%)	2.6 (13)	3 (16)	.004	0.58
**Nonjudgment**					
	Baseline	24 (6.66)	26 (10.5)	—	—
	Postintervention	29.7 (5.46)	30 (5)	—	—
	1-mo postintervention	28.3 (5.51)	29 (6)	—	—
	Δ at postintervention (%)	5.7 (24)	4 (15)	<.001	0.81
	Δ at 1-mo postintervention (%)	4.3 (18)	3 (12)	.003	0.60
**Personal distress**					
	Baseline	8.52 (4.43)	8 (5.5)	—	—
	Postintervention	6.96 (4.57)	7 (6)	—	—
	1-mo postintervention	6.74 (4.64)	6 (4.5)	—	—
	Δ at postintervention (%)	−1.56 (−18)	−1 (−12)	.03	0.43
	Δ at 1-mo postintervention (%)	−1.78 (−21)	−2 (−25)	.03	0.46
**Empathic concern**					
	Baseline	21.5 (5.02)	22 (6)	—	—
	Postintervention	22.1 (3.47)	22 (5.5)	—	—
	1-mo postintervention	21.6 (3.96)	22 (6)	—	—
	Δ at postintervention (%)	0.6 (2.8)	0 (0)	.81	0.07
	Δ at 1-mo postintervention (%)	0.1 (0.5)	0 (0)	.81	0.04
**Perspective taking**					
	Baseline	20.3 (4.57)	21 (4.5)	—	—
	Postintervention	21.2 (4.31)	21 (5.5)	—	—
	1-mo postintervention	21.7 (4.31)	22 (5)	—	—
	Δ at postintervention (%)	0.9 (4)	0 (0)	.18	0.26
	Δ at 1-mo postintervention (%)	1.4 (7)	1 (5)	.07	0.43

^a^Not applicable.

^b^Δ: change between baseline and stated time point.

^c^GAD-7: Generalized Anxiety Disorder-7.

^d^PHQ-2: Patient Health Questionnaire-2.

**Figure 4:**
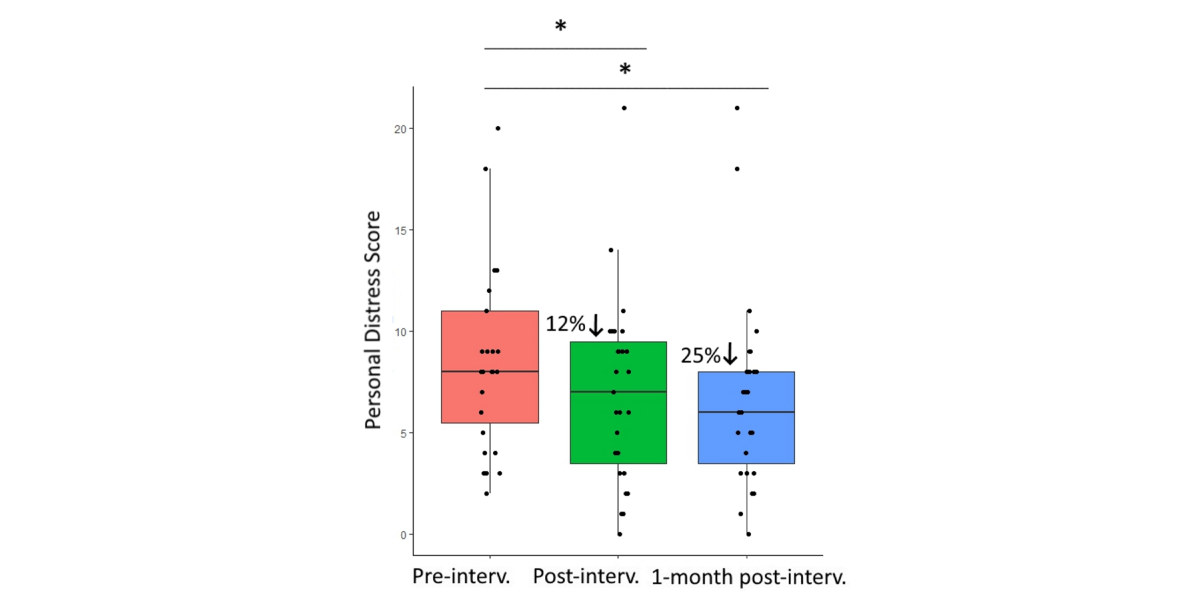
Box and whisker plots at baseline, at post-intervention, and 1 month after intervention completion, for personal distress scores. Significance level is denoted by asterisks: *P=.05.

Finally, Wilcoxon signed rank tests showed a 12% reduction in personal distress scores from baseline to postintervention (W_26_=217; *P*=.03; *r*=0.43) and a 25% reduction at 1 month after the end of the intervention (W_26_=200; *P*=.03; *r*=0.46; Figure 4).

#### Correlations Between Burnout and Other Outcomes

Table 3 shows the correlations between the 2 burnout dimensions of cynicism and emotional exhaustion and the other outcomes at each assessment point. There were significant correlations between burnout and anxiety at baseline and postintervention (cynicism: *r*≥0.56; *P*≤.03 and emotional exhaustion: *r*≥0.53; *P*≤.004) but not 1 month after the end of the intervention. Burnout scores were also correlated with nonreactivity scores at baseline and postintervention (cynicism: *r*≥0.39; *P*≤.04 and emotional exhaustion: *r*≥−0.40; *P*≤.04) but not at follow-up. Emotional exhaustion score was also correlated with self-compassion score only at baseline (*r*=−0.39; *P*≤.043). We did not observe any other correlations (*r*≤|0.37|; *P*≥|.06|).

**Table 3:** Correlations between burnout scores and other outcomes at baseline, postintervention, and 1-month postintervention.

Variables		Baseline	Postintervention	1-mo postintervention
**GAD-7^a^** **×** **cynicism**				
	Value	0.56	0.41	0.24
	*P* value	.002	.03	.22
**GAD-7** **×** **emotional exhaustion**				
	Value	0.60	0.53	0.32
	*P* value	<.001	.004	.99
**PHQ-2^b^** **×** **cynicism**				
	Value	0.25	0.12	0.11
	*P* value	.21	.56	.60
**PHQ-2** **×** **emotional exhaustion**				
	Value	0.17	0.08	0.21
	*P* value	.39	.70	.28
**IU^c^ × cynicism**				
	Value	0.37	0.02	0.16
	*P* value	.06	.91	.44
**IU** **×** **emotional exhaustion**				
	Value	0.15	0.36	0.12
	*P* value	.44	.07	.55
**Self-compassion** **×** **cynicism**				
	Value	−0.22	−0.16	−0.05
	*P* value	.27	.44	.80
**Self-compassion** **×** **emotional exhaustion**				
	Value	−0.39	−0.17	−0.03
	*P* value	.04	.41	.87
**Nonreactivity** **×** **cynicism**				
	Value	−0.39	−0.69	−0.35
	*P* value	.04	<.001	.07
**Nonreactivity** **×** **emotional exhaustion**				
	Value	−0.40	−0.49	−0.35
	*P* value	.04	.01	.07
**Nonjudgment** **×** **cynicism**				
	Value	−0.34	−0.13	−0.17
	*P* value	.09	.52	.41
**Nonjudgment** **×** **emotional exhaustion**				
	Value	−0.37	−0.02	−0.06
	*P* value	.06	.90	.76
**Personal distress** **×** **cynicism**				
	Value	0.10	0.08	0.34
	*P* value	.63	.67	.10
**Personal distress** **×** **emotional exhaustion**				
	Value	0.06	0.15	0.23
	*P* value	.77	.45	.24
**Empathic concern** **×** **cynicism**				
	Value	−0.07	−0.01	−0.24
	*P* value	.75	.95	.23
**Empathic concern** **×** **emotional exhaustion**				
	Value	0.27	0.24	0.14
	*P* value	.17	.24	.48
**Perspective taking** **×** **cynicism**				
	Value	−0.17	−0.09	−0.10
	*P* value	.38	.67	.62
**Perspective taking** **×** **emotional exhaustion**				
	Value	−0.03	−0.25	−0.15
	*P* value	.85	.21	.46

^a^GAD-7: Generalized Anxiety Disorder-7.

^b^PHQ-2: Patient Health Questionnaire-2.

^c^IU: intolerance of uncertainty.

## Study 2

### Overview

The results of study 1 provided evidence that physicians reported lower cynicism and emotional exhaustion at postintervention assessments compared to baseline. This was also the case for reduced anxiety, intolerance of uncertainty, and personal distress and increased self-compassion and mindfulness after the intervention. No difference was observed for depression and 2 subdimensions of empathy (ie, empathic concern and perspective taking) across time. This lack of change may be attributed to participants reporting low levels of depression and high levels of empathy at baseline.

To confirm the validity of these initial findings from study 1, we conducted a preregistered replication study. We used that same intervention but delivered it via a free smartphone-based platform called Unwinding by Sharecare (Behavioral Health team at Sharecare), which allows a progression of 7 daily modules of brief didactic; 1 summary at the end; and access to further features, such as experience-based MT, app-triggered check-ins, guided meditations (5-15 minutes), and clinician life stories.

Similar to study 1, participants completed web-based surveys at 3 time points: baseline, postintervention, and 1 month later as follow-up. To be more inclusive and expand to a broader range of clinicians, we recruited physicians and nurse practitioners, who provide direct care and medication prescriptions to their patients.

The same primary and secondary outcomes were included as in study 1, with the exception of the Interpersonal Reactivity Index, which was removed due to its lack of appropriateness for physicians, as indicated by the results of study 1.

In addition, based on the strong reduction of anxiety observed in study 1, we aimed to explore the effects of the intervention on further variables that have been suggested to be relevant for the onset and maintenance of anxiety, such as worry [76], sleep disturbances [77], and difficulties in emotion regulation [78].

We evaluated the following hypotheses:

- Hypothesis 1: there will be a main effect of time on cynicism. Physicians and nurse practitioners will report lower scores for cynicism at postintervention assessments compared to baseline.
- Hypothesis 2: there will be a main effect of time on the other variables. Physicians and nurse practitioners will report lower scores for emotional exhaustion, anxiety, depression, intolerance of uncertainty, worry, sleep disturbances, and difficulties in emotion regulation and higher scores for nonreactivity, nonjudging of inner experiences, and self-compassion at postintervention assessments compared to baseline.
- Hypothesis 3: cynicism and emotional exhaustion will positively correlate with anxiety.

### Methods

#### Ethical Considerations

Similar to study 1, this study was approved by the Brown University Institutional Review Board (protocol 2022003296) and followed the Declaration of Helsinki. The same consent process was used: participants were directed to the informed consent form on Qualtrics, and after selecting “yes” (with a waiver of documentation), they completed the web-based survey. Data were anonymized with alphanumeric codes, and the final database used only these codes. Likewise, participants were compensated with a US $25 Amazon gift card for the completion of each survey (US $75 for 3 surveys). The study was preregistered at ClinicalTrials.gov (NCT06145425), except for the exploratory outcome of difficulties in emotion regulation that we added later.

#### Participants

The same study design, recruitment process, and inclusion and exclusion criteria were used as in study 1, except for the additional recruitment of nurse practitioners. To be more inclusive, we recruited physicians and nurse practitioners, who are equally skilled and knowledgeable in providing direct care and medication prescriptions to their patients.

#### Intervention

The same intervention was delivered via the smartphone app Unwinding by Sharecare, which included 7 daily modules of MT, a final summary, app-triggered check-ins, guided meditations (5-15 minutes), and clinician life stories. Participants were encouraged to explore other app features; however, this was optional. Similar to study 1, participants completed 1 module per day at their own pace, with check-in messages from the project director every 15 days to address technical issues and encourage engagement.

#### Engagement

Data related to program engagement, as measured by the number of modules completed, were obtained directly from the Unwinding by Sharecare app.

#### Outcome Measures

The same approach was used as described in study 1: participants completed web-based surveys at 3 time points: baseline, postintervention, and 1 month later as follow-up. The same outcomes were included as described in study 1, with the following exceptions: the Interpersonal Reactivity Index was removed because the results of study 1 showed that this measure might not be appropriate for physicians. The Patient-Reported Outcomes Measurement Information System (PROMIS), the Penn State Worry Questionnaire, and the Difficulties in Emotion Regulation Scale were added as secondary outcomes to explore the effects of the intervention on sleep disturbances, worry, and difficulties in emotion regulation, respectively.

PROMIS was used to measure worry-related sleep disturbances. Five items from PROMIS were preselected based on their direct assessment of worry interfering with sleep [79]. Items were rated on a 5-point Likert scale (1=“not at all” and 5=“very much”). Scores range from 5 to 25, with higher scores indicating more sleep disturbances and greater sleep-related impairment [79,80].

Penn State Worry Questionnaire is a 16-item questionnaire to assess worry on a 5-point Likert scale (1=“not at all typical of me” and 5=“very typical of me”). Scores range from 16 to 80, with higher total scores indicating increased levels of worry [81].

Difficulties in Emotion Regulation Scale is a 36-item questionnaire used to assess emotion regulation challenges on a 5-point Likert scale (1=“almost never” and 5=“almost always”). Scores range from 36 to 180, with higher total scores indicating increased greater emotion regulation difficulty [82].

#### Statistical Analysis

The same data analysis approach described in study 1 was used.

### Results

#### Participants

**Figure 5:**
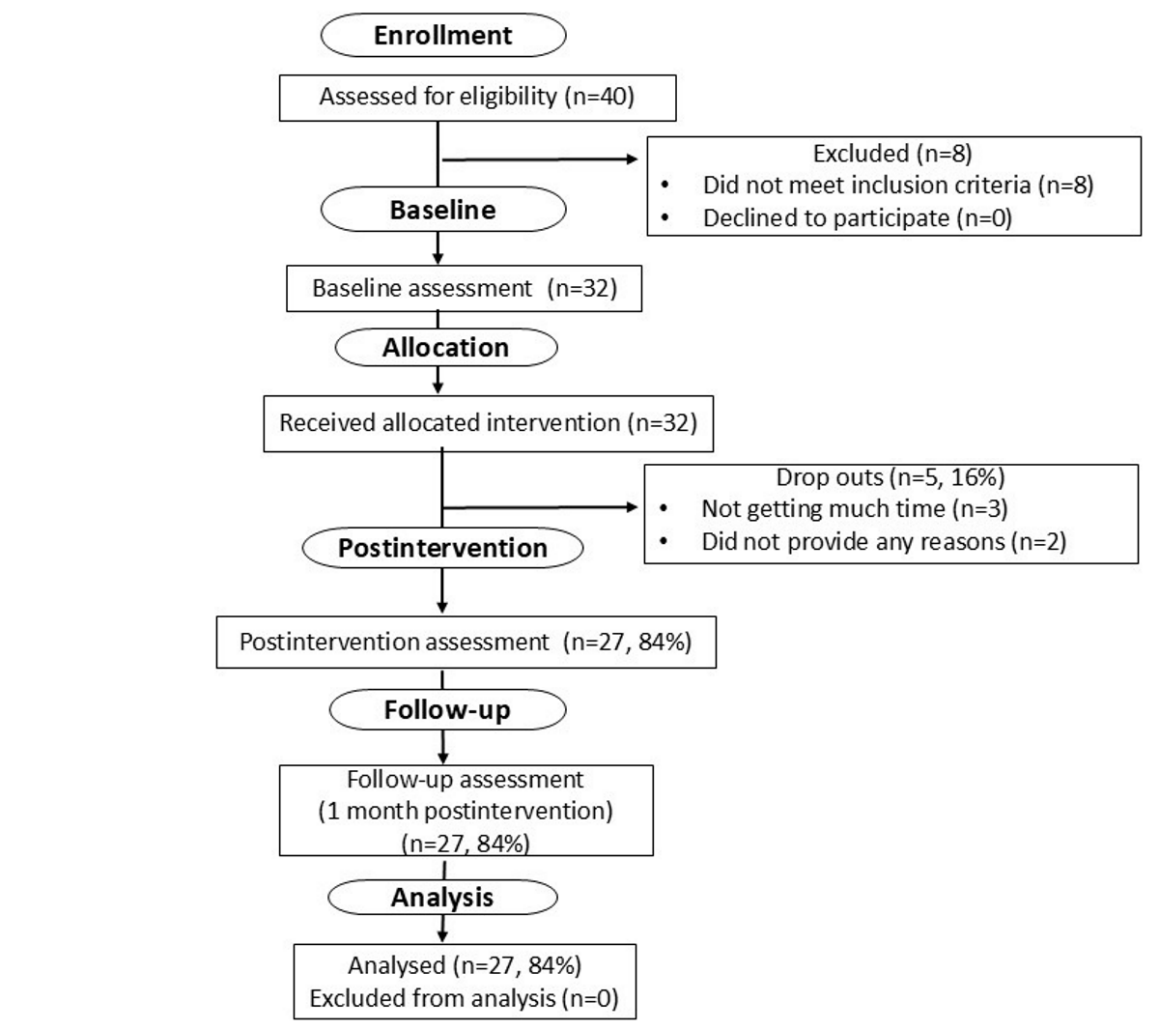
CONSORT (Consolidated Standards of Reporting Trials) flow diagram of study procedures of study 2.

A total of 40 physicians and 10 nurse practitioners replied to our recruitment message, and 39 (78%) participants (29 physicians and 10 nurse practitioners) met the eligibility criteria and consented to participate between June 2023 and May 2024. Out of this group who received the allocated intervention, of the N = 39 participants, 29 (74%, n=20, 69% physicians and n=9, 90% nurse practitioners) completed the 3-time point assessments and were included in the final analysis (Figure 5). The participant population comprised 23 women and 6 men who worked in health care for an average of 16.4 (SD 10.1) years. The average age was 49.6 (SD 9.8) years (Table 4).

**Table 4:** Demographic characteristics of study 2.

			Physicians+nurse practitioners (n=29)		Physicians (n=20)		Nurse practitioners (n=9)
Age (y), mean (SD)			49.6 (9.8)		49.8 (10.9)		49.1 (6.9)
**Gender, n (%)**							
	Men	6 (21)		5 (25)		1 (11)	
	Women	23 (79)		15 (75)		8 (89)	
**Race and ethnicity, n (%)**							
	Asian	3 (10)		3 (15)		0 (0)	
	Black or African American	2 (7)		1 (5)		1 (11)	
	Hispanic, Latinx, or Spanish	0 (0)		0 (0)		0 (0)	
	White	24 (83)		16 (80)		8 (89)	
**Work status, n (%)**							
	Employed full time	27 (93)		18 (90)		9 (100)	
	Employed part time	2 (7)		2 (10)		0 (0)	
Working duration in health care (y), mean (SD)			16.41 (10.14)		14.85 (8.83)		19.89 (11.86)
**Medical specialty, n (%)**							
	Emergency medicine	3 (10.2)		2 (10)		1 (11)	
	Pediatrics	6 (21)		2 (10)		4 (45)	
	Oncology	1 (3.4)		0 (0)		1 (11)	
	Palliative medicine	2 (7)		2 (10)		0 (0)	
	Family medicine	2 (7)		1 (5)		1 (11)	
	Internal medicine	4 (14)		4 (20)		0 (0)	
	Nephrology	1 (3.4)		1 (5)		0 (0)	
	Hospitality	1 (3.4)		1 (5)		0 (0)	
	Psychiatry	1 (3.4)		0 (0)		1 (11)	
	Infectious disease	1 (3.4)		1 (5)		0 (0)	
	Obstetrics-gynecology	3 (10.2)		3 (15)		0 (0)	
	Endocrinology	1 (3.4)		1 (5)		0 (0)	
	Clinical immunology	1 (3.4)		1 (5)		0 (0)	
	Physical medicine	1 (3.4)		1 (5)		0 (0)	
	Orthopedics	1 (3.4)		0 (0)		1 (11)	

#### Engagement

At posttreatment, 100% (29/29) of the participants completed the 7-module program; only 3 (10%) participants did not listen to the summary at the end. The average time taken by participants to complete the program was 23.8 (SD 18.7) days.

#### Primary and Secondary Outcomes

Multimedia Appendix 1 shows the outcome scores at each assessment point compared with baseline. Friedman ANOVA tests indicated significant changes over time for cynicism (*χ^2^*_2_=13.1; *P*=.001; W=0.23), emotional exhaustion (*χ^2^*_2_=2.39; *P*=.30; W=0.04), anxiety (*χ^2^*_2_=14.6; *P*<.001; W=0.25), intolerance of uncertainty (*χ^2^*_2_=15.6; *P*<.001; W=0.27), self-compassion (*χ^2^*_2_=22.2; *P*<.001; W=0.38), nonreactivity (*χ^2^*_2_=24.3; *P*<.001; W=0.42), and nonjudgment of inner experiences (*χ^2^*_2_=10.3; *P*=.006; W=0.18). Friedman ANOVA tests revealed significant changes over time for worry (*χ^2^*_2_=7.53; *P*=.02; W=0.13), sleep disturbances (*χ^2^*_2_=7.07; *P*=.03; W=0.122), and difficulties in emotion regulation (*χ^2^*_2_=15.4; *P*<.001; W=0.27). No significant change across the 3 time points was observed for depression (*χ^2^*_2_=1.77; *P*=.41; W=0.03).

**Figure 6:**
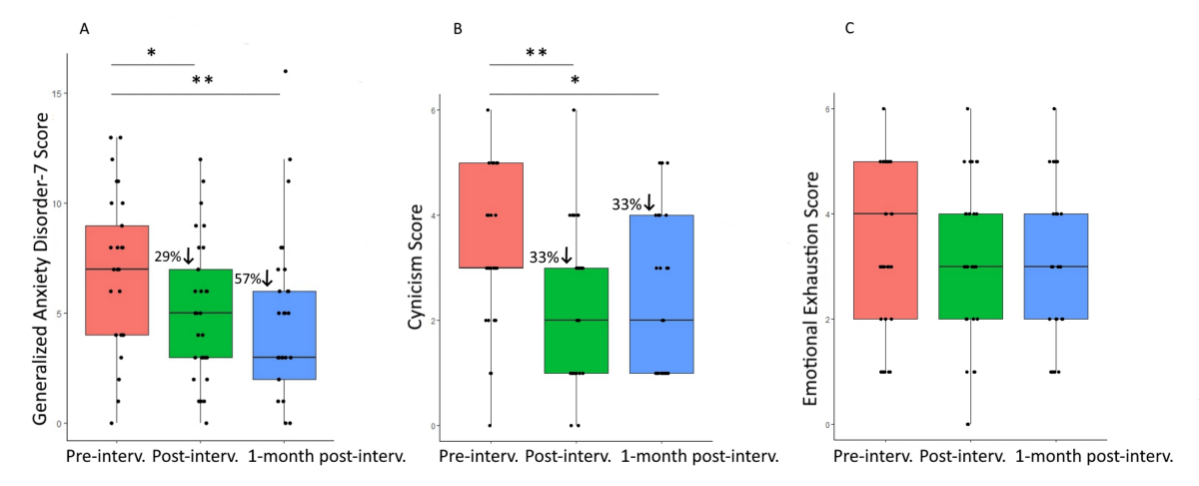
Box and whisker plots at baseline, at postintervention, and 1 month after intervention completion for (A) Generalized Anxiety Disorder-7 scores, (B) cynicism scores, and (C) emotional exhaustion scores from the Maslach Burnout Inventory in 29 physicians and nurse practitioners. *P=.05, **P=.01.

Post hoc Wilcoxon signed rank tests revealed 33% reduction of cynicism at both postintervention (W_28_=184; *P*=.008; *r*=0.58) and 1 month after the end of the intervention (W_28_=196; *P*=.04; *r*=0.45) and 29% reduction of anxiety scores from baseline to postintervention (W_28_=317; *P*=.01; *r*=0.49) and 57% reduction 1 month later (W_28_=340; *P*=.006; *r*=0.59; Figure 6). We observed stronger results when we analyzed only the data from physicians: a significant change across the 3 time points for cynicism (*χ^2^*_2_=16.3; *P*<.001; W=0.41) and a 50% reduction in scores from baseline to postintervention (W_19_=105; *P*=.003; *r*=0.81) and 33% reduction 1 month later (W_19_=128; *P*=.02; *r*=0.58). Likewise, we observe similar results for anxiety over time (*χ^2^*_2_=10.5; *P*=.005; W=0.26) and 33% reduction from baseline to postintervention (W_19_=150; *P*=.04; *r*=0.51) and 60% reduction 1 month later (W_19_=165; *P*=.02; *r*=0.63).

Wilcoxon signed rank tests found 12% reduction of intolerance of uncertainty scores from baseline to postintervention (W_26_=338; *P*=.003; *r*=0.57) and 8% reduction 1 month after the end of the intervention (W_26_=310; *P*<.001; *r*=0.66) and 21% increase in self-compassion scores from baseline to postintervention (W_28_=32.5; *P*<.001; *r*=0.69) and 27% increase 1 later (W_28_=2; *P*<.001; *r*=0.84). We observed similar results with data collected only from physicians: significant changes across the 3 time points for intolerance of uncertainty (*χ^2^*_2_=9.24; *P*=.01; W=0.23) and for self-compassion (*χ^2^*_2_=15.5; *P*<.001; W=0.39). Intolerance of uncertainty scores reduced by 22% at postintervention (W_19_=162; *P*=.01; *r*=0.59) and by 24% one month later (W_19_=158; *P*=.003; *r*=0.72) and self-compassion scores increased by 31% at postintervention (W_19_=9.5; *P*=.001; *r*=0.74) and 38% one month later (W_19_=1.5; *P*<.001; *r*=0.82).

**Figure 7:**
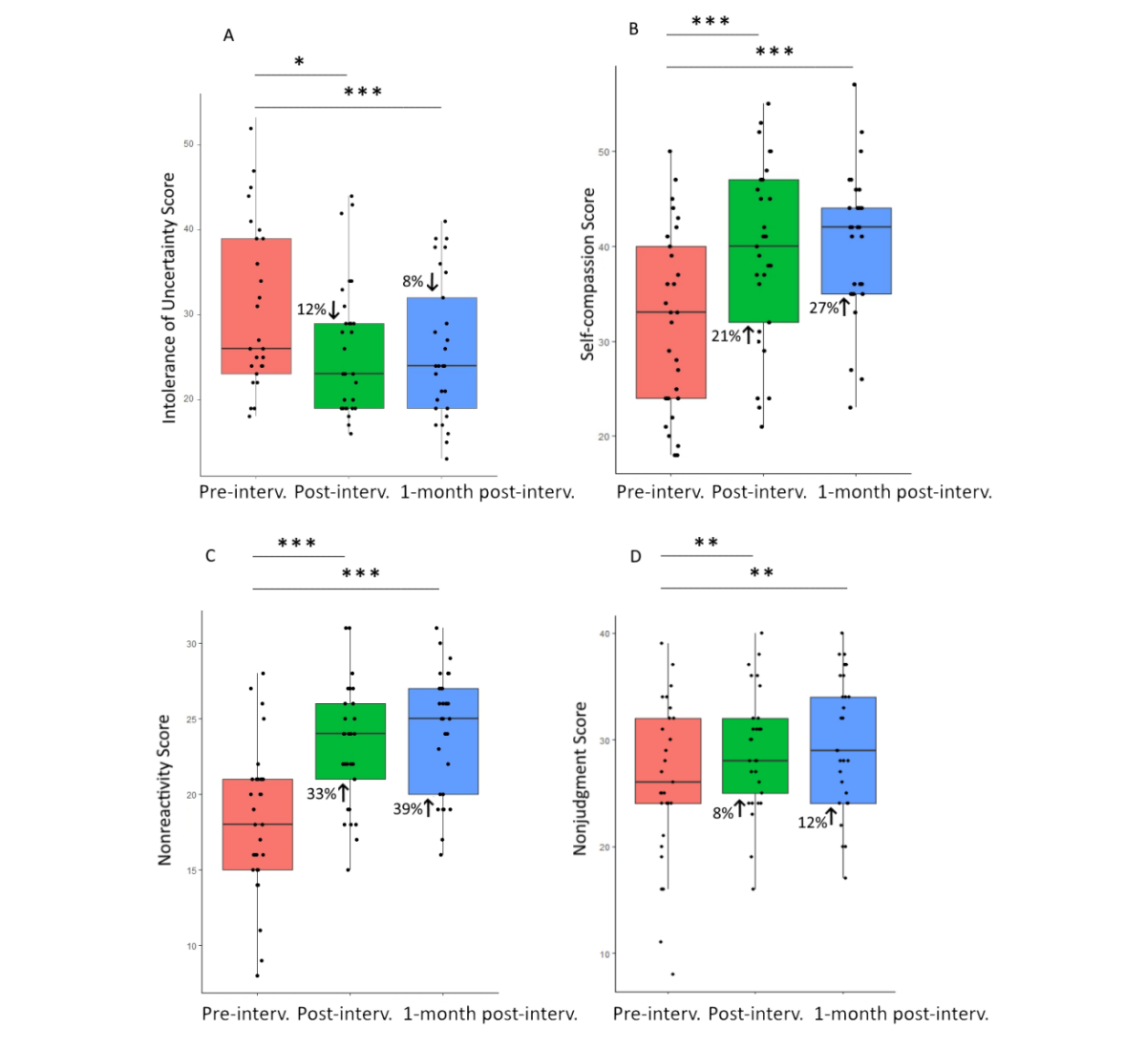
Box and whisker plots at baseline, at postintervention, and 1 month after intervention completion for (A) intolerance of uncertainty scores, (B) self-compassion scores, and (C) nonreactivity toward inner experiences scores in 29 physicians and nurse practitioners. *P=.05, **P=.01,***P=.001.

Wilcoxon signed rank tests also found 33% increase in nonreactivity scores from baseline to postintervention (W_28_=38.5; *P*<.001; *r*=0.72) and 39% increase 1 month after the end of the intervention (W_28_=9; *P*<.001; *r*=0.83) and 8% increase in nonjudgment scores at postintervention (W_28_=60.5; *P*=.009; *r*=0.50) and 12% increase 1 month later (W_28_=38.5; *P*=.003; *r*=0.62; Figure 7). We observed similar results with data only from physicians: a significant change across the 3 time points for nonreactivity (*χ^2^*_2_=18.8; *P*<.001; W=0.47) and for nonjudgment (*χ^2^*_2_=7.10; *P*=.03; W=0.18) and 28% increase in nonreactivity scores at postintervention (W_19_=21; *P*=.003; *r*=0.71) and 39% increase 1 month later (W_19_=4; *P*<.001; *r*=0.85) and 10% increase in nonjudgment scores at postintervention (W_19_=40.5; *P*=.08; *r*=0.43) and 12% increase 1 month later (W_19_=19.5; *P*=.02; *r*=0.62).

**Figure 8:**
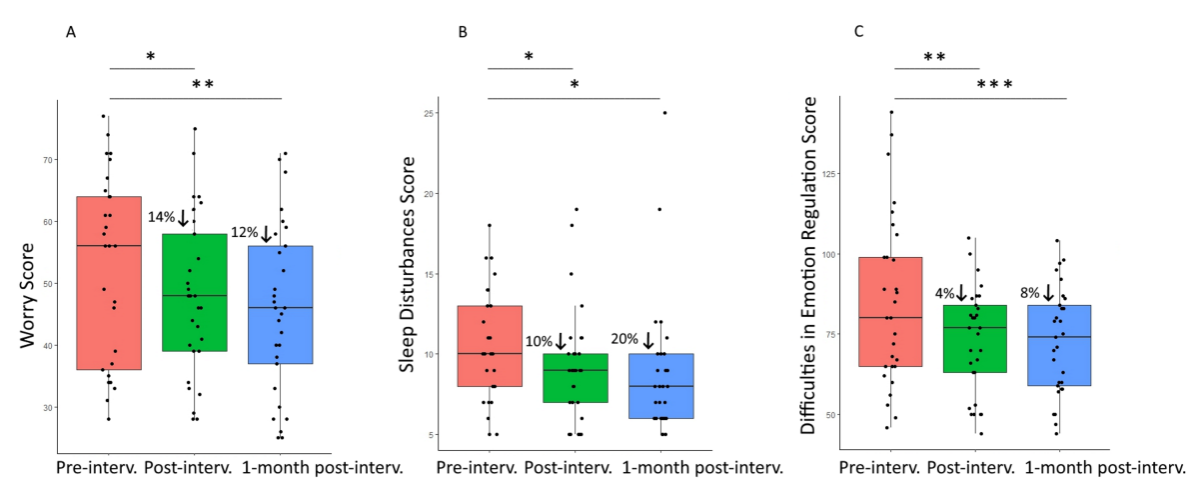
Box and whisker plots at baseline, at postintervention, and 1 month after intervention completion for (A) worry scores, (B) sleep disturbances scores, and (C) difficulties in emotion regulation scores in 29 physicians and nurse practitioners. *P=.05, **P=.01,***P=.001.

Finally, Wilcoxon signed rank tests revealed 14% reduction in worry scores from baseline to postintervention (W_28_=247; *P*=.04; *r*=0.40) and 12% reduction 1 month after the end of the intervention (W_28_=319; *P*=.006; *r*=0.58), 10% reduction in sleep disturbances at postintervention (W_28_=283; *P*=.04; *r*=.42) and 20% reduction 1 month later (W_28_=268; *P*=.01; *r*=0.53), and 4% reduction in difficulties in emotion regulation scores at postintervention (W_28_=353; *P*=.005; *r*=0.54) and 8% reduction 1 month later (W_28_=392; *P*<.001; *r*=0.70; Figure 8). We observe less strong results with data only from physicians: a trend change across the 3 time points for worry (*χ^2^*_2_=5.30; *P*=.07; W=0.13) and significant changes for sleep (*χ^2^*_2_=7.53; *P*=.02; W=0.188) and emotion regulation (*χ^2^*_2_=9.29; *P*=.009; W=0.23). We only observed 21% reduction in worry at 1 month after the end of the intervention (W_19_=168; *P*=.01; *r*=0.66) but not at postintervention (W_19_=116; *P*=.09; *r*=0.37). We found 22% reduction in sleep disturbances scores from baseline to postintervention (W_19_=156; *P*=.02; *r*=0.54) and 30% reduction 1 month later (W_19_=152; *P*=.01; *r*=0.64) and 8% reduction in difficulties in emotion regulation at postintervention (W_19_=176; *P*=.01; *r*=0.59) and 11% reduction 1 month later (W_19_=192; *P*=.04; *r*=0.73).

#### Correlations Between Burnout and Other Outcomes

Table 5 shows the correlations between the 2 burnout dimensions of cynicism and emotional exhaustion and the other outcomes at each assessment point. There were significant correlations between anxiety and burnout at baseline and 1 month after the end of the intervention (cynicism: *r*≥0.39; *P*≤.04 and emotional exhaustion: *r*≥0.58; *P*≤.001) but not right after the intervention. Burnout were correlated also with self-compassion scores (cynicism: *r*≥−0.42; *P*≤.02 and emotional exhaustion: *r*=−0.56; *P*=.002), intolerance of uncertainty scores (cynicism: *r*≥0.37; *P*≤.05 and emotional exhaustion: *r*≥0.40; *P*≤.03), and depression (cynicism: *r*=0.41; *P*=.03 and emotional exhaustion: *r*=0.41; *P*=.03). Burnout scores were also correlated with worry (cynicism: *r*≥0.45; *P*≤.01 and emotional exhaustion: *r*≥0.51; *P*≤.004) and emotion regulation (cynicism: *r*≥0.46; *P*≤.01 and emotional exhaustion: *r*=0.40; *P*=.03). Cynicism scores were also correlated with nonjudgment at baseline (*r*=−0.49; *P*=.006) and with sleep after the intervention (*r*=0.38; *P*=.04). Emotional exhaustion scores were also correlated with nonreactivity scores only at baseline (*r*=−0.56; *P*=.002). We did not observe any other correlations (*r*≤|0.36|; *P*≥|.054|).

**Table 5:** Correlations between anxiety and burnout scores at baseline, postintervention, and 1-month postintervention.

Variables		Baseline	Postintervention	1-mo postintervention
**GAD-7^a^ × cynicism**				
	Value	0.56	0.41	0.24
	*P* value	.002	.03	.22
**GAD-7 × emotional exhaustion**				
	Value	0.60	0.53	0.32
	*P* value	<.001	.004	.10
**PHQ-2^b^ × cynicism**				
	Value	0.22	0.05	0.41
	*P* value	.25	.81	.03
**PHQ-2** **×** **emotional exhaustion**				
	Value	0.35	0.23	0.41
	*P* value	.06	.24	.03
**IU^c^ × cynicism**				
	Value	0.52	0.28	0.37
	*P* value	.004	.13	.05
**IU** **×** **emotional exhaustion**				
	Value	0.41	0.45	0.4
	*P* value	.03	.01	.03
**Self-compassion** **×** **cynicism**				
	Value	−0.52	−0.42	−0.62
	*P* value	.004	.002	<.001
**Self-compassion** **×** **emotional exhaustion**				
	Value	−0.35	−0.17	−0.56
	*P* value	.06	.37	.002
**Nonreactivity** **×** **cynicism**				
	Value	−0.27	−0.20	−0.3
	*P* value	.15	.29	.11
**Nonreactivity** **×** **emotional exhaustion**				
	Value	−0.56	−0.3	−0.26
	*P* value	.002	.12	.12
**Nonjudgment** **×** **cynicism**				
	Value	−0.49	−0.3	−0.34
	*P* value	.006	.11	.07
**Nonjudgment** **×** **emotional exhaustion**				
	Value	−0.25	−0.18	−0.34
	*P* value	.19	.35	.08
**Worry** **×** **cynicism**				
	Value	−0.49	−0.45	−0.64
	*P* value	.007	.01	.001
**Worry** **×** **emotional distress**				
	Value	0.51	0.32	0.54
	*P* value	.004	.09	.003
**Difficulties in emotion regulation** **×** **cynicism**				
	Value	0.49	0.33	0.53
	*P* value	.01	.08	.003
**Difficulties in emotion regulation** **×** **emotional exhaustion**				
	Value	0.35	0.31	0.4
	*P* value	.07	.10	.03
**Sleep disturbances** **×** **cynicism**				
	Value	−0.22	−0.38	−0.25
	*P* value	.26	.04	.19
**Sleep disturbances** **×** **emotional exhaustion**				
	Value	−0.29	−0.2	0.36
	*P* value	.12	.30	.60

^a^GAD-7: Generalized Anxiety Disorder-7.

^b^PHQ-2: Patient Health Questionnaire-2.

^c^IU: intolerance of uncertainty.

## Discussion

### Principal Findings

To our knowledge, this is the first research to assess the efficacy of a digital MT program designed with input from clinicians specifically to address burnout. Two independent single-arm studies were conducted to evaluate its impact on physicians. In the first study, MT was delivered via podcast, while in the second study, it was administered through a free mobile app.

Results from both studies demonstrated that MT led to reductions in burnout, anxiety, intolerance of uncertainty, and personal distress, while also fostering increases in self-compassion and mindfulness, with effect sizes ranging from medium to large. In addition, the second study revealed decreases in worry, sleep disturbances, and difficulties in emotion regulation. However, no significant changes were observed over time for depression or perspective taking and empathic concern, potentially due to the already low levels of depression and high levels of perspective taking and empathic concern reported at baseline. Furthermore, significant positive correlations were found between burnout and anxiety at baseline in both studies, at postintervention in study 1, and at follow-up in study 2, thus confirming the relationship between these 2 variables.

### Reduction of Physician Burnout

Preliminary findings suggest that this training could be an efficacious, accessible way for busy physicians to combat burnout. Both studies showed that it reduced cynicism, with the first also reducing emotional exhaustion. This may be due to the focus of the intervention on individual well-being, particularly addressing the interpersonal aspect of burnout. However, individual-focused interventions alone may not fully address physician burnout, which often requires collaborative efforts at both the individual and organizational levels [33,83].

### Increase of Mindfulness and Self-Compassion

This study builds upon prior research demonstrating that in-person mindfulness-based interventions have been effective in reducing burnout and anxiety among physicians [37-40]. Importantly, we were able to replicate these findings using a digital program that is both free and less time consuming than the intensive in-person MT used in previous studies. This digital intervention may therefore offer a more feasible option for integration into the already hectic schedules of physicians. In addition, this intervention contributes to the growing field of digital therapeutics by demonstrating clinically meaningful effects [7,60,61,84].

Consistent with numerous studies, we found that MT increased nonreactivity and nonjudgmental attitudes toward thoughts, sensations, and emotions, while also enhancing self-compassion. Self-compassion has been theorized to provide self-care during times of distress, failure, and difficulty and to enable individuals to offer compassionate care to others. This concept comprises 3 interconnected components: mindfulness, self-kindness, and common humanity [46]. In practical terms, self-compassion can facilitate several key aspects: (1) recognizing and accepting feelings as they arise without suppression or avoidance (ie, mindfulness), (2) treating oneself with kindness and understanding (ie, self-kindness), and (3) acknowledging one’s own pain and distress as part of the broader human experience (ie, common humanity [46]).

Therefore, self-compassion may enable health care providers, who often struggle with perfectionism and self-criticism in high-stakes environments where errors can have grave consequences, to better navigate the challenges they face. Such feelings of inadequacy have been linked to anxiety, depression, and even suicidal ideation among medical students [85,86] and physicians [87]. For instance, interventions promoting self-compassion among athletes have proven successful in helping them manage concerns about mistakes, self-criticism, and rumination during setbacks and uncertainties, thereby improving both athletic performance and psychological well-being [88].

### Reduction of Personal Distress and Intolerance of Uncertainty

Regarding empathy, we only observed a significant reduction in personal distress during postintervention assessments. This finding aligns with existing literature, which has consistently reported that personal distress is the empathic component positively associated with burnout [89]. However, we did not observe any effects on the other 2 dimensions of empathy: perspective taking and empathic concern. This outcome may be due to a ceiling effect, as previous research with health care professionals has also reported [90]. The high baseline scores suggest that the chosen questionnaire may not have been sensitive enough to capture changes in these dimensions among physicians.

Although previous literature has shown a link between high intolerance of uncertainty and an increased risk or presence of physician burnout [24,91] and work-related stress and satisfaction [21,92], these 2 studies are the first to investigate the effects of MT on intolerance of uncertainty. We found a significant reduction with a large effect size. Recognizing uncertainty and developing coping strategies are crucial in the field of medicine and are recognized as essential clinical competencies to be fostered throughout medical education and training [93,94].

For example, when faced with clinical situations where outcomes are difficult to predict, physicians with high intolerance of uncertainty may experience excessive worry and anxiety about the consequences of their decisions. They may also struggle with self-doubt and insecurity regarding their professional competence, leading to a sense of being “stuck” in uncertainty and hesitancy in decision-making [95,96]. It is estimated that 17% of the excessive costs in medical care result from physicians’ anxiety related to uncertainty management, leading to increased tendencies to order further tests [97], failures to adhere to evidence-based guidelines [98], and fear of malpractice litigation and defensive practices [99].

### Reduction of Anxiety and Related Variables

Importantly, we observed that MT reduced anxiety by 40% postintervention, with moderate to large effect sizes. Furthermore, we confirmed the positive correlation between burnout and anxiety, although this correlation was not significant across all 3 time points in both studies. These results build upon previous findings regarding the relationship between burnout and anxiety [7].

Interestingly, MT also reduced variables previously associated with the onset and maintenance of anxiety, such as worry, sleep disturbances, and difficulties with emotion regulation. In previous research, app-based MT significantly reduced anxiety in individuals with generalized anxiety disorders [61] and improved sleep disturbances [84]. These effects were mediated by a reduction in worry [61,84]. MT appears to assist individuals in interrupting habitual worry patterns by fostering present-focused awareness of thoughts and emotions. Guiding physicians to observe and manage repetitive worry patterns rather than reacting to and reinforcing them could alleviate anxiety and burnout.

In addition, research indicates that being aware of one’s own emotions and regulating them in stressful work environments can not only reduce personal distress but also promote prosocial behavior [100,101]. For instance, when physicians encounter patients with sudden worsening symptoms, they need to manage their own discomfort to provide appropriate care for their patients considered distressed.

### Limitations

These studies offer several strengths and significant findings, such as a freely accessible training program designed for real-world application, preregistered outcomes, and the inclusion of a diverse range of medical specialties in the samples. However, they also have several limitations.

First, both studies are single-arm trials with relatively short follow-up periods (ie, 1 month after completing the training). To validate the efficacy of this program and ascertain its long-term effects, future studies using a waiting list or active control intervention with longer follow-up periods are warranted. These studies could also investigate the underlying psychological mechanisms.

Second, the study samples are relatively small and not fully representative, with predominantly White and female physician participants. While future studies with balanced representation across sexes are necessary for generalizability, it is worth noting that women are more likely to be affected by burnout and anxiety compared to men [102-104]. In addition, it is important to recognize that burnout affects not only physicians but also other health care professionals and students. Moving forward, it is critical to adapt and test interventions across a wider population within the health care system. Addressing clinician burnout early in professional development and acknowledging specific stressors in the learning environment are essential steps in mitigating its impact. In addition, both studies involved self-selection of participants, meaning that those who chose to participate were likely already predisposed to favor the program. This suggests that the intervention might have been particularly beneficial for individuals who were attracted to the mindfulness component of the program. As a result, we do not know whether the intervention would affect those who were less interested in mindfulness or less inclined to participate.

Finally, the impact of the intervention was assessed exclusively through self-report measures and individual-related variables, without considering the individual-organization relationship. As we have noted, both individual and organizational factors contribute to burnout. Similar to recent findings in the field [105], future research should incorporate measures such as work engagement, teamwork, and feelings of being valued by the organization, in addition to patient-related ecological outcomes. While collecting data on mortality rates, complication rates, and disease progression might be challenging across different health care institutions, other measures such as patient compliance, the strength of the patient–health care provider relationship, compassionate care, and testing and prescribing behaviors can be effectively implemented. Understanding these broader outcomes is crucial for developing effective interventions and strategies to address physician burnout and improve patient care and health outcomes.

### Conclusions

Despite these limitations, both studies demonstrated that physicians were open to trying a pragmatically designed and delivered digital MT program and could potentially benefit from it. They suggest that digital MT holds promise as an accessible, evidence-based tool for addressing burnout and related aspects of anxiety among physicians. However, it is important to note that causal claims cannot be definitively made until future randomized controlled studies are conducted. These proof-of-concept studies serve as an initial exploration of potential mechanisms and effect size calculations for larger randomized controlled trials.

Furthermore, this evidence underscores the need for both individual-focused and organizational-focused approaches to addressing burnout, and it highlights the importance of involving physicians in the design of new interventions to create effective and sustainable strategies for preventing or mitigating burnout and promoting professional well-being.

## Appendix

Multimedia Appendix 1 Descriptive statistics for primary and secondary outcomes (N=29 physicians+nurse practitioners).

## Abbreviations

MT: mindfulness training
PROMIS: Patient-Reported Outcomes Measurement Information System

## Data Availability

The datasets generated and analyzed during this study are available from the corresponding author on reasonable request.

## References

[1] Shanafelt TD, Boone S, Tan L, Dyrbye LN, Sotile W, Satele D, West CP, Sloan J, Oreskovich MR (2012). Burnout and satisfaction with work-life balance among US physicians relative to the general US population. Arch Intern Med.

[2] Shanafelt TD, Gradishar WJ, Kosty M, Satele D, Chew H, Horn L, Clark B, Hanley AE, Chu Q, Pippen J, Sloan J, Raymond M (2014). Burnout and career satisfaction among US oncologists. J Clin Oncol.

[3] Shanafelt TD, Hasan O, Dyrbye LN, Sinsky C, Satele D, Sloan J, West CP (2015). Changes in burnout and satisfaction with work-life balance in physicians and the general US working population between 2011 and 2014. Mayo Clin Proc.

[4] Shanafelt TD, West CP, Dyrbye LN, Trockel M, Tutty M, Wang H, Carlasare LE, Sinsky C (2022). Changes in burnout and satisfaction with work-life integration in physicians during the first 2 years of the COVID-19 pandemic. Mayo Clin Proc.

[5] (2019). Burn-out an "occupational phenomenon": international classification of diseases. World Health Organization.

[6] Maslach C, Leiter MP (2017). New insights into burnout and health care: strategies for improving civility and alleviating burnout. Med Teach.

[7] Roy A, Druker S, Hoge EA, Brewer JA (2020). Physician anxiety and burnout: symptom correlates and a prospective pilot study of app-delivered mindfulness training. JMIR Mhealth Uhealth.

[8] Melamed S, Ugarten U, Shirom A, Kahana L, Lerman Y, Froom P (1999). Chronic burnout, somatic arousal and elevated salivary cortisol levels. J Psychosom Res.

[9] Vela-Bueno A, Moreno-Jiménez B, Rodríguez-Muñoz A, Olavarrieta-Bernardino S, Fernández-Mendoza J, De la Cruz-Troca JJ, Bixler EO, Vgontzas AN (2008). Insomnia and sleep quality among primary care physicians with low and high burnout levels. J Psychosom Res.

[10] Weaver MD, Robbins R, Quan SF, O'Brien CS, Viyaran NC, Czeisler CA, Barger LK (2020). Association of sleep disorders with physician burnout. JAMA Netw Open.

[11] Ryan E, Hore K, Power J, Jackson T (2023). The relationship between physician burnout and depression, anxiety, suicidality and substance abuse: a mixed methods systematic review. Front Public Health.

[12] Shanafelt TD, Balch CM, Dyrbye L, Bechamps G, Russell T, Satele D, Rummans T, Swartz K, Novotny PJ, Sloan J, Oreskovich MR (2011). Special report: suicidal ideation among American surgeons. Arch Surg.

[13] Schernhammer ES, Colditz GA (2004). Suicide rates among physicians: a quantitative and gender assessment (meta-analysis). Am J Psychiatry.

[14] Sinsky CA, Brown RL, Stillman MJ, Linzer M (2021). COVID-related stress and work intentions in a sample of US health care workers. Mayo Clin Proc Innov Qual Outcomes.

[15] Han S, Shanafelt TD, Sinsky CA, Awad KM, Dyrbye LN, Fiscus LC, Trockel M, Goh J (2019). Estimating the attributable cost of physician burnout in the United States. Ann Intern Med.

[16] Dewa CS, Loong D, Bonato S, Trojanowski L (2017). The relationship between physician burnout and quality of healthcare in terms of safety and acceptability: a systematic review. BMJ Open.

[17] Salyers MP, Bonfils KA, Luther L, Firmin RL, White DA, Adams EL, Rollins AL (2017). The relationship between professional burnout and quality and safety in healthcare: a meta-analysis. J Gen Intern Med.

[18] Dyrbye LN, Varkey P, Boone SL, Satele DV, Sloan JA, Shanafelt TD (2013). Physician satisfaction and burnout at different career stages. Mayo Clin Proc.

[19] Southwick FS, Southwick SM (2018). The loss of a sense of control as a major contributor to physician burnout: a neuropsychiatric pathway to prevention and recovery. JAMA Psychiatry.

[20] Freudenberger HJ (2010). Staff burn‐out. J Soc Issues.

[21] Iannello P, Mottini A, Tirelli S, Riva S, Antonietti A (2017). Ambiguity and uncertainty tolerance, need for cognition, and their association with stress. A study among Italian practicing physicians. Med Educ Online.

[22] Begin AS, Hidrue M, Lehrhoff S, Del Carmen MG, Armstrong K, Wasfy JH (2022). Factors associated with physician tolerance of uncertainty: an observational study. J Gen Intern Med.

[23] Strout TD, Hillen M, Gutheil C, Anderson E, Hutchinson R, Ward H, Kay H, Mills GJ, Han PK (2018). Tolerance of uncertainty: a systematic review of health and healthcare-related outcomes. Patient Educ Couns.

[24] Cooke GP, Doust JA, Steele MC (2013). A survey of resilience, burnout, and tolerance of uncertainty in Australian general practice registrars. BMC Med Educ.

[25] Klimecki O, Singer T, Oakley B, Knafo A, Madhavan G, Wilson DS (2012). Empathic distress fatigue rather than compassion fatigue? Integrating findings from empathy research in psychology and social neuroscience. Pathological Altruism.

[26] Burns DD, Nolen-Hoeksema S (1992). Therapeutic empathy and recovery from depression in cognitive-behavioral therapy: a structural equation model. J Consult Clin Psychol.

[27] Del Canale S, Louis DZ, Maio V, Wang X, Rossi G, Hojat M, Gonnella JS (2012). The relationship between physician empathy and disease complications: an empirical study of primary care physicians and their diabetic patients in Parma, Italy. Acad Med.

[28] Hojat M, Louis DZ, Markham FW, Wender R, Rabinowitz C, Gonnella JS (2011). Physicians' empathy and clinical outcomes for diabetic patients. Acad Med.

[29] Pereira L, Figueiredo-Braga M, Carvalho IP (2016). Preoperative anxiety in ambulatory surgery: the impact of an empathic patient-centered approach on psychological and clinical outcomes. Patient Educ Couns.

[30] Rakel DP, Hoeft TJ, Barrett BP, Chewning BA, Craig BM, Niu M (2009). Practitioner empathy and the duration of the common cold. Fam Med.

[31] Mercer SW, Reynolds WJ (2002). Empathy and quality of care. Br J Gen Pract.

[32] Shanafelt TD, West C, Zhao X, Novotny P, Kolars J, Habermann T, Sloan J (2005). Relationship between increased personal well-being and enhanced empathy among. J Gen Intern Med.

[33] West CP, Dyrbye LN, Erwin PJ, Shanafelt TD (2016). Interventions to prevent and reduce physician burnout: a systematic review and meta-analysis. Lancet.

[34] Ruotsalainen JH, Verbeek JH, Mariné A, Serra C (2015). Preventing occupational stress in healthcare workers. Cochrane Database Syst Rev.

[35] Luberto CM, Wasson RS, Kraemer KM, Sears RW, Hueber C, Cotton S (2017). Feasibility, acceptability, and preliminary effectiveness of a 4-week mindfulness-based cognitive therapy protocol for hospital employees. Mindfulness (N Y).

[36] Cascales-Pérez ML, Ferrer-Cascales R, Fernández-Alcántara M, Cabañero-Martínez MJ (2021). Effects of a mindfulness-based programme on the health- and work-related quality of life of healthcare professionals. Scand J Caring Sci.

[37] Goodman MJ, Schorling JB (2012). A mindfulness course decreases burnout and improves well-being among healthcare providers. Int J Psychiatry Med.

[38] Gracia Gozalo RM, Ferrer Tarrés JM, Ayora Ayora A, Alonso Herrero M, Amutio Kareaga A, Ferrer Roca R (2019). Application of a mindfulness program among healthcare professionals in an intensive care unit: effect on burnout, empathy and self-compassion. Med Intensiva (Engl Ed).

[39] Krasner MS, Epstein RM, Beckman H, Suchman AL, Chapman B, Mooney CJ, Quill TE (2009). Association of an educational program in mindful communication with burnout, empathy, and attitudes among primary care physicians. JAMA.

[40] Martín-Asuero A, García-Banda G (2010). The Mindfulness-based Stress Reduction program (MBSR) reduces stress-related psychological distress in healthcare professionals. Span J Psychol.

[41] Kabat-Zinn J (1991). Full Catastrophe Living: Using the Wisdom of Your Body and Mind to Face Stress, Pain, and Illness.

[42] Lindsay EK, Creswell JD (2017). Mechanisms of mindfulness training: Monitor and Acceptance Theory (MAT). Clin Psychol Rev.

[43] Watson T, Walker O, Cann R, Varghese AK (2021). The benefits of mindfulness in mental healthcare professionals. F1000Res.

[44] Benzo RP, Anderson PM, Bronars C, Clark M (2018). Mindfulness for healthcare providers: the role of non-reactivity in reducing stress. Explore (NY).

[45] Wasson RS, Barratt C, O'Brien WH (2020). Effects of mindfulness-based interventions on self-compassion in health care professionals: a meta-analysis. Mindfulness (N Y).

[46] Neff KD (2003). Self-compassion: an alternative conceptualization of a healthy attitude toward oneself. Self Identity.

[47] Neff KD, Hsieh YP, Dejitterat K (2005). Self-compassion, achievement goals, and coping with academic failure. Self Identity.

[48] Sinclair S, Beamer K, Hack TF, McClement S, Raffin Bouchal S, Chochinov HM, Hagen NA (2017). Sympathy, empathy, and compassion: a grounded theory study of palliative care patients' understandings, experiences, and preferences. Palliat Med.

[49] Duarte J, Pinto-Gouveia J, Cruz B (2016). Relationships between nurses' empathy, self-compassion and dimensions of professional quality of life: a cross-sectional study. Int J Nurs Stud.

[50] Kemper KJ, McClafferty H, Wilson PM, Serwint JR, Batra M, Mahan JD, Schubert CJ, Staples BB, Schwartz A, Pediatric Resident Burnout-Resilience Study Consortium (2019). Do mindfulness and self-compassion predict burnout in pediatric residents?. Acad Med.

[51] Montero-Marin J, Zubiaga F, Cereceda M, Piva Demarzo MM, Trenc P, Garcia-Campayo J (2016). Burnout subtypes and absence of self-compassion in primary healthcare professionals: a cross-sectional study. PLoS One.

[52] Olson K, Kemper KJ, Mahan JD (2015). What factors promote resilience and protect against burnout in first-year pediatric and medicine-pediatric residents?. J Evid Based Complementary Altern Med.

[53] Babenko O, Mosewich AD, Lee A, Koppula S (2019). Association of physicians' self-compassion with work engagement, exhaustion, and professional life satisfaction. Med Sci (Basel).

[54] Scheepers RA, Emke H, Epstein RM, Lombarts KM (2020). The impact of mindfulness-based interventions on doctors' well-being and performance: a systematic review. Med Educ.

[55] Shapiro SL, Astin JA, Bishop SR, Cordova M (2005). Mindfulness-based stress reduction for health care professionals: results from a randomized trial. Int J Stress Manag.

[56] Valley M, Stallones L (2018). A thematic analysis of health care workers' adoption of mindfulness practices. Workplace Health Saf.

[57] Swensen S, Kabcenell A, Shanafelt T (2016). Physician-organization collaboration reduces physician burnout and promotes engagement: the mayo clinic experience. J Healthc Manag.

[58] Brewer J (2019). Mindfulness training for addictions: has neuroscience revealed a brain hack by which awareness subverts the addictive process?. Curr Opin Psychol.

[59] Brewer JA, Mallik S, Babuscio TA, Nich C, Johnson HE, Deleone CM, Minnix-Cotton CA, Byrne SA, Kober H, Weinstein AJ, Carroll KM, Rounsaville BJ (2011). Mindfulness training for smoking cessation: results from a randomized controlled trial. Drug Alcohol Depend.

[60] Mason AE, Jhaveri K, Cohn M, Brewer JA (2018). Testing a mobile mindful eating intervention targeting craving-related eating: feasibility and proof of concept. J Behav Med.

[61] Roy A, Hoge EA, Abrante P, Druker S, Liu T, Brewer JA (2021). Clinical efficacy and psychological mechanisms of an app-based digital therapeutic for generalized anxiety disorder: randomized controlled trial. J Med Internet Res.

[62] Gleichgerrcht E, Decety J (2013). Empathy in clinical practice: how individual dispositions, gender, and experience moderate empathic concern, burnout, and emotional distress in physicians. PLoS One.

[63] Gleichgerrcht E, Decety J (2014). The relationship between different facets of empathy, pain perception and compassion fatigue among physicians. Front Behav Neurosci.

[64] Neumann M, Edelhäuser F, Tauschel D, Fischer MR, Wirtz M, Woopen C, Haramati A, Scheffer C (2011). Empathy decline and its reasons: a systematic review of studies with medical students and residents. Acad Med.

[65] Rowe JP, Shores LR, Mott BW, Lester JC (2010). Integrating learning and engagement in narrative-centered learning environments. Proceedings of the 10th International Conference on Intelligent Tutoring Systems.

[66] West CP, Dyrbye LN, Sloan JA, Shanafelt TD (2009). Single item measures of emotional exhaustion and depersonalization are useful for assessing burnout in medical professionals. J Gen Intern Med.

[67] West CP, Dyrbye LN, Satele DV, Sloan JA, Shanafelt TD (2012). Concurrent validity of single-item measures of emotional exhaustion and depersonalization in burnout assessment. J Gen Intern Med.

[68] Maslach C, Jackson SE, Leiter MP, Zalaquett CP, Wood RJ (1997). Maslach burnout inventory. Evaluating Stress: A Book of Resources.

[69] Spitzer RL, Kroenke K, Williams JB, Löwe B (2006). A brief measure for assessing generalized anxiety disorder: the GAD-7. Arch Intern Med.

[70] Kroenke K, Spitzer RL, Williams JB (2001). The PHQ-9: validity of a brief depression severity measure. J Gen Intern Med.

[71] Carleton RN, Norton MA, Asmundson GJ (2007). Fearing the unknown: a short version of the Intolerance of Uncertainty Scale. J Anxiety Disord.

[72] Raes F, Pommier E, Neff KD, Van Gucht D (2011). Construction and factorial validation of a short form of the Self-Compassion Scale. Clin Psychol Psychother.

[73] Baer RA, Smith GT, Lykins E, Button D, Krietemeyer J, Sauer S, Walsh E, Duggan D, Williams JM (2008). Construct validity of the five facet mindfulness questionnaire in meditating and nonmeditating samples. Assessment.

[74] Davis MH (1983). Measuring individual differences in empathy: evidence for a multidimensional approach. J Pers Soc Psychol.

[75] Cohen J (1992). A power primer. Psychol Bull.

[76] Roemer L, Salters K, Raffa SD, Orsillo SM (2005). Fear and avoidance of internal experiences in GAD: preliminary tests of a conceptual model. Cogn Ther Res.

[77] Cox RC, Olatunji BO (2020). Sleep in the anxiety-related disorders: a meta-analysis of subjective and objective research. Sleep Med Rev.

[78] Cisler JM, Olatunji BO, Feldner MT, Forsyth JP (2010). Emotion regulation and the anxiety disorders: an integrative review. J Psychopathol Behav Assess.

[79] Yu L, Buysse DJ, Germain A, Moul DE, Stover A, Dodds NE, Johnston KL, Pilkonis PA (2011). Development of short forms from the PROMIS™ sleep disturbance and sleep-related impairment item banks. Behav Sleep Med.

[80] Buysse DJ, Yu L, Moul DE, Germain A, Stover A, Dodds NE, Johnston KL, Shablesky-Cade MA, Pilkonis PA (2010). Development and validation of patient-reported outcome measures for sleep disturbance and sleep-related impairments. Sleep.

[81] Meyer TJ, Miller ML, Metzger RL, Borkovec TD (1990). Development and validation of the Penn State worry questionnaire. Behav Res Ther.

[82] Gratz KL, Roemer L (2008). Multidimensional assessment of emotion regulation and dysregulation: development, factor structure, and initial validation of the difficulties in emotion regulation scale. J Psychopathol Behav Assess.

[83] Carrau D, Janis JE (2021). Physician burnout: solutions for individuals and organizations. Plast Reconstr Surg Glob Open.

[84] Gao M, Roy A, Deluty A, Sharkey KM, Hoge EA, Liu T, Brewer JA (2022). Targeting anxiety to improve sleep disturbance: a randomized clinical trial of app-based mindfulness training. Psychosom Med.

[85] Eley DS, Bansal V, Leung J (2020). Perfectionism as a mediator of psychological distress: implications for addressing underlying vulnerabilities to the mental health of medical students. Med Teach.

[86] Enns MW, Cox BJ, Sareen J, Freeman P (2001). Adaptive and maladaptive perfectionism in medical students: a longitudinal investigation. Med Educ.

[87] Craiovan PM (2014). Correlations between perfectionism, stress, psychopathological symptoms and burnout in the medical field. Procedia Soc Behav Sci.

[88] Mosewich AD, Crocker PR, Kowalski KC, Delongis A (2013). Applying self-compassion in sport: an intervention with women athletes. J Sport Exerc Psychol.

[89] Delgado N, Delgado J, Betancort M, Bonache H, Harris L (2023). What is the link between different components of empathy and burnout in healthcare professionals? A systematic review and meta-analysis. Psychol Res Behav Manag.

[90] Boellinghaus I, Jones FW, Hutton J (2012). The role of mindfulness and loving-kindness meditation in cultivating self-compassion and other-focused concern in health care professionals. Mindfulness.

[91] Kuhn G, Goldberg R, Compton S (2009). Tolerance for uncertainty, burnout, and satisfaction with the career of emergency medicine. Ann Emerg Med.

[92] Bovier PA, Perneger TV (2007). Stress from uncertainty from graduation to retirement--a population-based study of Swiss physicians. J Gen Intern Med.

[93] Englander R, Cameron T, Ballard AJ, Dodge J, Bull J, Aschenbrener CA (2013). Toward a common taxonomy of competency domains for the health professions and competencies for physicians. Acad Med.

[94] Simpson JG, Furnace J, Crosby J, Cumming AD, Evans PA, Friedman Ben David M, Harden RM, Lloyd D, McKenzie H, McLachlan JC, McPhate GF, Percy-Robb IW, MacPherson SG (2002). The Scottish doctor--learning outcomes for the medical undergraduate in Scotland: a foundation for competent and reflective practitioners. Med Teach.

[95] Lally J, Cantillon P (2014). Uncertainty and ambiguity and their association with psychological distress in medical students. Acad Psychiatry.

[96] Simpkin AL, Schwartzstein RM (2016). Tolerating uncertainty - the next medical revolution?. N Engl J Med.

[97] Allison JJ, Kiefe CI, Cook EF, Gerrity MS, Orav EJ, Centor R (1998). The association of physician attitudes about uncertainty and risk taking with resource use in a Medicare HMO. Med Decis Making.

[98] Ghosh AK (2004). On the challenges of using evidence-based information: the role of clinical uncertainty. J Lab Clin Med.

[99] Benbassat J, Pilpel D, Schor R (2001). Physicians' attitudes toward litigation and defensive practice: development of a scale. Behav Med.

[100] Eisenberg N, Fabes RA, Murphy B, Karbon M, Maszk P, Smith M, O'Boyle C, Suh K (1994). The relations of emotionality and regulation to dispositional and situational empathy-related responding. J Pers Soc Psychol.

[101] Ochsner KN, Gross JJ (2005). The cognitive control of emotion. Trends Cogn Sci.

[102] Catuzzi JE, Beck KD (2014). Anxiety vulnerability in women: a two-hit hypothesis. Exp Neurol.

[103] Hoff T, Lee DR (2021). Burnout and physician gender: what do we know?. Med Care.

[104] Lyubarova R, Salman L, Rittenberg E (2023). Gender differences in physician burnout: driving factors and potential solutions. Perm J.

[105] Epstein RM, Marshall F, Sanders M, Krasner MS (2022). Effect of an intensive mindful practice workshop on patient-centered compassionate care, clinician well-being, work engagement, and teamwork. J Contin Educ Health Prof.

