# Supplementary material for: Digital Mindfulness Training for Burnout Reduction in Physicians: Clinician-Driven Approach
Source: JMIR Form Res. 2025 Jan 24;9:e63197. doi: 10.2196/63197 (PMC11806274; doi:10.2196/63197)
Supplement: Multimedia Appendix 1 [file formative_v9i1e63197_app1.docx]

**Table 1.** Descriptive statistics for primary and secondary outcomes (N=29 physicians+nurse practitioners).

|  | | Values, mean (SD) | Values, median (IQR) | *P* value | Effect sizes (*r*) |
| --- | --- | --- | --- | --- | --- |
| **Cynicism** | | | | | |
|  | Baseline | 3.34 (1.45) | 3 (2) | —^a^ | — |
|  | Postintervention | 2.24 (1.55) | 2 (2) | — | — |
|  | 1-mo postintervention | 2.52 (1.53) | 2 (3) | — | — |
|  | Δ^b^ at postintervention (%) | −1.1 (−33) | −1 (−33) | .008 | 0.58 |
|  | Δ at 1-mo postintervention (%) | −0.82 (−25) | −1 (−33) | .04 | 0.45 |
| **Emotional exhaustion** | | | | | |
|  | Baseline | 3.45 (1.55) | 4 (3) | — | — |
|  | Postintervention | 3.07 (1.56) | 3 (2) | — | — |
|  | 1-mo postintervention | 3.10 (1.54) | 3 (2) | — | — |
|  | Δ at postintervention (%) | −0.4 (−11) | −1 (−25) | .19 | 0.26 |
|  | Δ at 1-mo postintervention (%) | −0.35 (−10) | −1 (−33) | .19 | 0.30 |
| **GAD-7^c^** | | | | | |
|  | Baseline | 6.90 (3.51) | 7 (5) | — | — |
|  | Postintervention | 5.07 (3.17) | 5 (4) | — | — |
|  | 1-mo postintervention | 4.69 (3.75) | 3 (4) | — | — |
|  | Δ at postintervention (%) | −1.83 (−27) | −2 (−29) | .01 | 0.49 |
|  | Δ at 1-mo postintervention (%) | −2.21 (−32) | −4 (−57) | .006 | 0.59 |
| **PHQ-2^d^** | | | | | |
|  | Baseline | 0.66 (0.97) | 0 (1) | — | — |
|  | Postintervention | 0.52 (0.69) | 0 (1) | — | — |
|  | 1-mo postintervention | 0.48 (0.87) | 0 (1) | — | — |
|  | Δ at postintervention (%) | −0.14 (−21) | 0 (0) | .75 | 0.08 |
|  | Δ at 1-mo postintervention (%) | −0.17 (−26) | 0 (0) | .75 | 0.25 |
| **Intolerance of uncertainty** | | | | | |
|  | Baseline | 31.1 (10.4) | 26 (16) | — | — |
|  | Postintervention | 26 (7.95) | 23 (10) | — | — |
|  | 1-mo postintervention | 25.7 (8.30) | 24 (13) | — | — |
|  | Δ at postintervention (%) | −5.1 (16) | −3 (−12) | .003 | 0.57 |
|  | Δ at 1-mo postintervention (%) | −5.4 (17) | −2 (−8) | .002 | 0.66 |
| **Self-compassion** | | | | | |
|  | Baseline | 32.1 (9.56) | 33 (16) | — | — |
|  | Postintervention | 39.3 (9.54) | 40 (15) | — | — |
|  | 1-mo postintervention | 40.3 (7.54) | 42 (9) | — | — |
|  | Δ at postintervention (%) | 7.2 (22) | 7 (21) | <.001 | 0.69 |
|  | Δ at 1-mo postintervention (%) | 8.2 (26) | 9 (27) | <.001 | 0.84 |
| **Nonreactivity** | | | | | |
|  | Baseline | 18.3 (4.9) | 18 (6) | — | — |
|  | Postintervention | 23.2 (4.03) | 24 (5) | — | — |
|  | 1-mo postintervention | 24.2 (4.05) | 25 (7) | — | — |
|  | Δ at postintervention (%) | 4.9 (27) | 6 (33) | <.001 | 0.72 |
|  | Δ at 1-mo postintervention (%) | 5.9 (32) | 7 (39) | <.001 | 0.83 |
| **Nonjudgment** | | | | | |
|  | Baseline | 26.1 (7.46) | 26 (8) | — | — |
|  | Postintervention | 29 (5.6) | 28 (7) | — | — |
|  | 1-mo postintervention | 29.5 (6.26) | 29 (11) | — | — |
|  | Δ at postintervention (%) | 2.9 (11) | 2 (8) | .009 | 0.50 |
|  | Δ at 1-mo postintervention (%) | 3.4 (12) | 3 (12) | .003 | 0.62 |
| **Worry** | | | | | |
|  | Baseline | 52.1 (15.3) | 56 (28) | — | — |
|  | Postintervention | 47.9 (12.9) | 48 (19) | — | — |
|  | 1-mo postintervention | 45.8 (13.7) | 46 (19) | — | — |
|  | Δ at postintervention (%) | −4.2 (−8) | −8 (−14) | .04 | 0.40 |
|  | Δ at 1-mo postintervention (%) | −6.3 (−12) | −10 (−12) | .006 | 0.58 |
| **Sleep disturbances** | | | | | |
|  | Baseline | 11 (4) | 10 (5) | — | — |
|  | Postintervention | 9.14 (3.62) | 9 (3) | — | — |
|  | 1-mo postintervention | 8.76 (4.26) | 8 (4) | — | — |
|  | Δ at postintervention (%) | −1.86 (−17) | −1 (−10) | .04 | 0.41 |
|  | Δ at 1-mo postintervention (%) | −2.24 (−20) | −2 (−20) | .01 | 0.53 |
| **Difficulties in emotion regulation** | | | | | |
|  | Baseline | 85 (26.5) | 80 (34) | — | — |
|  | Postintervention | 73 (16.6) | 77 (21) | — | — |
|  | 1-mo postintervention | 72.8 (16.7) | 74 (25) | — | — |
|  | Δ at postintervention (%) | −12 (−14) | −3 (−4) | .005 | 0.54 |
|  | Δ at 1-mo postintervention (%) | −12.2 (−14) | −6 (−8) | <.001 | 0.70 |
